# Surface-assembled non-noble metal nanoscale Ni-colloidal thin-films as efficient electrocatalysts for water oxidation†

**DOI:** 10.1039/c9ra07388d

**Published:** 2019-11-14

**Authors:** Noor-Ul-Ain Babar, Ayesha Saddiqa, Laraib Nisar, Syeda Robina Gilani, Khurram Saleem Joya

**Affiliations:** Department of Chemistry, University of Engineering and Technology (UET) GT Road 54890 Lahore Pakistan khurram.joya@gmail.com; Institute of Chemical Sciences, Bahauddin Zakariya University Multan-60800 Pakistan

## Abstract

A highly operative and inexpensive water oxidation scheme using an efficient nanoscale electrocatalyst is vastly demanded for optimum H_2_ production, CO_2_ reduction, and has attracted increased attention for chemical energy conversion. We present here a simple route to make efficient electrocatalytic colloidal nanoparticles of nickel out of mere metal ions in a simple borate buffer system. The simple and annealed Ni-colloidal nanoparticles (Ni-CNPs) resulted in a facile transformation into ultrafine films, which further activated the catalysts, while initiating OER just at the overpotential *η* = 250 mV (1.48 V *vs.* RHE) under benign conditions. They also showed high porosity and favorable kinetics while displaying impressive Tafel slopes of just 51 mV dec^−1^, and a high TOF value of 0.79 s^−1^ at 0.35 V was observed for Ni-CNPs/FTO_500_. These electrocatalysts also showed long-term stability during the bulk water electrolysis experiment conducted for a continuous 20 hours without notable catalytic degradation, which ensures their economic benefits. The electrochemical data, CVs, kinetic study, short-term durability, extended catalytic stability, SEM analysis, and other supporting data provide compelling evidence that these non-precious, metal-based, electroactive, catalytic, colloidal thin-films (simple and annealed) with nanoscale morphological attributes presented promising catalytic performance under the conditions used herein.

## Abstract 2

Highly applied and accessible electrocatalytic system derived from simple Ni-colloids has been explored to facilely derive kinetically sluggish water oxidation reaction. Ni-catalysts also present well-balanced kinetics of OER and high durability.

## Graphical abstract

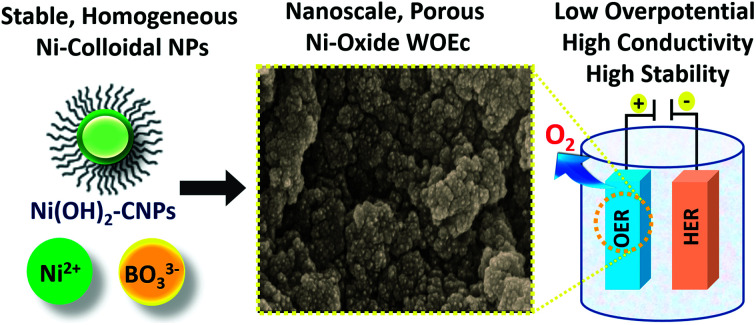

## Introduction

Owing to the extensive consumption of conventional fuels and their consequent adverse effects on the environment, the ultimate exploration of a sustainable energy system is the only vital way to satisfy the future global energy demand, which is expected to increase two-fold by 2050 and up to three-fold by 2100.^1^ This is also associated with inventing cleaner energy resources to resolve environmental problems due to incremental CO_2_ accumulation caused by the burning of carbon-based fossil fuels in automobiles and power generation houses.^2,3^ Regarding these challenges, photoelectrochemical and electrochemical water splitting to generate O_2_ and H_2_ is a promising way to produce cheap, abundant and eco-friendly fuel with zero emissions.^4^ Water splitting is a two-step reaction that includes the oxygen evolution reaction (OER) and hydrogen evolution reaction (HER), and is very challenging from a technical viewpoint. Of these two half-reactions, the oxidation of water to release four electrons and make molecular oxygen is kinetically more demanding,^5^ and considered to be a bottleneck due to its uphill thermodynamics and transfer of multiple electrons.^6^ On the other hand, the very sluggish OER is intensely imperative as it is firmly associated with the number of energy renewable systems such as solar cells, metal–air batteries and photoelectric cell (PEC)-based water splitting.^7,8^ Therefore, the main challenge is to develop an efficient water oxidation assembly and a super electrocatalyst that should be low-cost, geologically abundant, operating under moderate conditions and synthesized *via* facile methods.^9,10^

A large number of electrocatalyst materials has been developed during the last two decades. Mono/multinuclear homo-heterogeneous catalysts for efficient water oxidation catalysis have been discovered, providing in-depth mechanistic details of the reaction.^10^ The benchmark precious metal catalysts, such as RuO_2_, IrO_2,_ and Pd-based thin film materials, show excellent catalytic activity in both acidic and basic media.^1,7^ However, their widespread commercial applications are largely restricted due to high cost and scarceness.^11,12^ Therefore, it is desirable to rationally design and develop prevalent and low-cost alternatives that are efficient and effective.^13,14^

Transition metal oxides are known to meet the said criteria, and extensive efforts have been made to develop transition metal derived simple^15–18^ and mixed metal oxides and hydroxides,^19,20^ chalcogenides,^21^ phosphate-^22^ and boride^23^-based electrocatalytic materials. More specifically, the metal oxides/hydroxide-based electrocatalysts comprising iron group elements (such as nickel, cobalt, copper, iron and manganese, their nanoparticles and thin films) have been tremendously reported for OER under alkaline conditions.^24–29^ Among them, the nickel and cobalt oxides/hydroxides (being inexpensive, ductile,^14^ and thermally stable) have emerged as interesting catalytic candidates for environmentally friendly water splitting catalysis.^30^ Furthermore, the nano-structuring of the electrocatalyst is also getting renowned, as it is thought to enhance the electrochemically active area of the catalyst for the efficient adsorption of various intermediates, ultimate O–O bond formation and subsequent release of molecular oxygen from the catalytic layer and better permanence during OER.^24,25^

Atomically dispersed iron embedded onto the nitrogen-doped graphene showed remarkable water oxidation activity under alkaline conditions.^29^ Also, the insertion of electroactive materials having high active sites has also been shown to bring desirable catalytic features in the targeted catalytic materials for successful implications.^26–29^ In this respect, various synthetic strategies for the fabrication of nanoscale transition metals (and more specifically, Ni-based water oxidation electrocatalysts) have been put forward including the chemical reduction method,^25^ controlled current anodization method,^24*b*^ electrodeposition method,^6,7^ hydrothermal method, and emulsion method.^31–33^ In the pursuit of this, Joya *et al.* prepared highly active nickel nanoclusters (NiNCs) following the chemical reduction method, which is capable of oxidizing water at 1.51 V (*vs.* RHE) and showing remarkable stability during long-term application.^25^ However, the fabrication of NiNCs needs too much chemical inputs and prolonged methods. A series of nano-scale electrodeposited Ni-Ci,^31^ Co-Ci,^7^ and Ag-Ci^34^ based thin film electrocatalytic assemblages have been synthesized in a bicarbonate/CO_2_ (pH = 6.8–7) system for water oxidation catalysis. The catalysts were highly active in the cost-effective carbonate/bicarbonate electrolyte, exhibiting remarkable stability during long-term water electrolysis. Following that, in a separate study, Li *et al.* introduced a highly active Fe-based electrocatalyst by electrodeposition from a CO_2_-enriched bicarbonate system containing Fe^2+^ ions. The Fe-Ci catalyst was highly active and durable.^35^ The catalysts were deposited from inexpensive electrolyte solutions having a near-neutral pH and are cost-effective, but cause a deposition at the cathode surface as well. To avoid this issue, expensive polymeric separators are needed that can limit their implications, although the materials discussed above showed optimum water catalysis efficiency under the employed conditions.

However, the use of too much complicated chemical environments, extensive synthetic strategies, and sophisticated instrumental setups led to the continuous quest for replacing those complex synthetic methods with some economical, straightforward and facile strategies that can be worthwhile for large scale applications. Therefore, it is believed that substantial efforts are still needed in developing a highly efficient catalyst material with an optimum activity using low-cost materials and highly applied green methods involving fewer chemicals and simpler setups.^24,33^

**Scheme 1:**
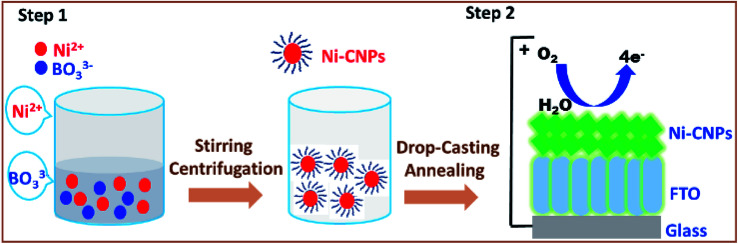
Illustrating the formation of nanoscale nickel colloidal nanoparticles obtainable from metal solutions and borate buffer (step 1). Further, Ni-CNPs are coated onto the FTO substrate (with or without applying post-annealing operations) to be employed as highly conductive, durable and low overpotential water oxidation electrocatalysts.

In this regard, we present here a very easy, time-efficient, inexpensive and simple stratagem to fabricate oxide-hydroxide colloidal nanoparticles of nickel of the type Ni(OH)_*x*_/BO_3_^3−^ and their thin films, preferably on simple anodic substrates (which are inert for water splitting catalysis) to be employed as an efficient water oxidation electrocatalyst (Scheme 1 and S1†). These nanoparticles can advantageously be prepared in just a few minutes from metal ion solutions with simple buffer solutions. Furthermore, the surface deposited thin-films of the thus-obtained oxide-hydroxide colloidal nanoparticles are easily obtained on the conductor, such as the fluorine-doped tin oxide (FTO) *via* simple drop-casting with or without post-annealing at the low-temperature (Table S1†). It was observed that, after applying the heat treatments at low temperatures (250–500 °C), the catalysts showed interesting morphological features and enhanced electrocatalytic activity for the water oxidation reaction. Moreover, the method presents the simplistic approach of inserting carbon and boron content in the catalytic layer, and thus making them into efficient, robust and flexible nanomaterials. The metal–O–B network that might have formed can further enhance the catalytic activity and boost the stability for water conversion to molecular oxygen. Furthermore, we extensively studied and evaluated the electrochemical activity, kinetics and stability of the Ni-colloidal nanoparticle (Ni-CNPs) derived heterogeneous nano-electrocatalysts for the oxygen evolution reaction, following a modified protocol that included optimum electrochemical parameters under the employed conditions,^36^ as represented in Scheme S2.† The easily measured parameters were adapted to compare the electrocatalytic efficiency of the thin-film electrocatalyst. The relevant figure of merit includes the onset overpotential for OER, Tafel slope measurements and the overpotential needed to achieve the current density of 10 mA cm^−2^ during the continuous controlled current electrolysis (CCE) experiment for water splitting. For the sake of applicability, long-term controlled-potential electrolysis (CPE) experiments were also conducted. The electrocatalytic efficiency of the FTO electrode without catalytic coating was also investigated for comparison.

## Experimental section

### Materials and methods

Analytical grade reagents were purchased from Aldrich and used as received without any purification step, unless otherwise mentioned. FTO-coated glass slides (resistivity < 7–10 ohms per sq, the transmittance at 550 nm ≥ 86%, FTO film thickness 1800–2000 Å, haze ≤ 2%) were obtained from Aldrich and cleaned well prior to use. All glassware and electrochemical cells were cleaned following a previously described method.^1,6,7^

#### Synthesis of colloidal nanoparticles

The colloidal nanoparticle (CNPs) dispersion of nickel was prepared using the optimized amounts of metal ion solution and buffers (borate) of specific concentration and pH. All synthetic conditions to prepare the CNPs were carefully optimized *via* cross experimentations. In this pursuit, 100 μL of a 0.2 M borate buffer solution (pH ≈ 10) was added to a 1 mL clear solution of 0.1 M Ni^2+^ ions, and the mixture was stirred gently for a while and allowed to stand for a few minutes until the solution turned slightly opaque, which was the clear indication for the formation of colloids of the type Ni(OH)_2_ and Ni(OH)_2_/BO_3_^3−^. Finally, the colloids were filtered using microfilter syringes to obtain homogeneously sized NPs (further details for the preparation of the colloidal NPs are described in ESI, Table S1†).

#### Fabrication of working electrode

In order to prepare anode materials decorated with a thin-film water oxidation nano-electrocatalyst for catalyzing OER, the colloidal nanoparticles obtained as described above are directly deposited onto the conductor surface, employing a simplistic drop-casting approach to obtain a homogeneous catalytic layer. For this purpose, a 15 to 20 μL aliquot of the as-prepared metal colloidal NPs are deposited onto the conducting side of the FTO surface and air-dried. In another experiment, to see the effect of post-heat treatments on the structural and morphological attributes and electrochemical activity of the catalytic film, the anode adorned with a thin-film nano-electrocatalyst (following deposition) was annealed at 250 °C and 500 °C for 1 hour by keeping inside a furnace. The catalytic films (un-annealed and annealed) obtained as described above were placed directly in an electrochemical cell as an anode for water catalysis, specifically OER studies, under the employed electrochemical conditions.

#### Electrochemical characterization

All electrochemical investigations (such as cyclic voltammetry (CV), controlled potential electrolysis and controlled current electrolysis) were performed in a standard three-electrode configuration pyrex glass cell on a computer-controlled potentiostat (Autolab PG-Stat 10), using the nano-electrocatalyst films supported on FTO directly as the working electrode, platinum wire as a counter electrode and a saturated silver/silver chloride (sat. Ag/AgCl) and saturated calomel electrode (SCE) as the reference electrodes. The FTO slides were cut to the suitable size, pre-treated by sonication in ultrapure water, isopropanol, and acetone (15 minutes with each one) and dried in an oven at about 70 °C for 10–20 minutes. The counter electrode was cleaned by immersion in a 20% solution of nitric acid, and rinsed many times with distilled water before placement into the electrochemical cell. Considering the relatively superior catalytic performance of the Ni-based catalysts in alkaline conditions, all electrochemical data were collected under alkaline conditions.^36^ Electrolyte solutions were prepared using ultrapure water and NaOH pellets (99.99%). The cyclic voltammograms were conducted in a 0.1 M aq. NaOH electrolyte solution having pH ≈ 13 at the scan rate of 5, 20 and 50 mV s^−1^, unless otherwise mentioned. The overpotential was calculated according to the formula, overpotential [*η*] = *E*_RHE_ − 1.23 V. Electrochemical impedance spectroscopy was used to evaluate the solution resistance (*R*_ct_) and charge transfer resistance (*R*_ct_) on the electrode–electrolyte interphase, fitting a simplified Randles circuit using a similar electrochemical set-up. EIS was acquired in the frequency range from 0.1 Hz to 100 kHz at 1.58 V *vs.* RHE applied potential. Electrochemically accessible catalytic sites were evaluated by integrating the area under the reduction peak of Ni^2+/3+^ redox couple under the optimal potential range. To study the intrinsic efficiency of the nano-electrocatalysts, the electrochemical parameters (such as the exchange current density (*J*°) and turnover frequency (TOF)) were evaluated using the standard eqn (S1) and (S2).†

#### Scale conversion

All potentials reported here were converted into reversible hydrogen electrode (RHE), according to the Nernst equation^1^ given below.*E*_RHE_ = *E*_REF_ + *E*^0^_REF_ + 0.059 (pH)Here, *E*^0^_REF_ for Ag/AgCl is 0.197 V and for SCE is 0.241 V.

#### IR corrections

For all electrochemical data (cyclic voltammetry, CPE, CCE, and Tafel plot calculations), the IR corrections were made manually using the given equation:^1^*E*_actual_ − IR = *E*_corrected_

#### Tafel slope calculations from linear polarization curve of CV scan

The Tafel slopes were calculated by considering the current density under the various potentials directly obtained from the CV data, and manually correcting the data for the IR drop. The study was acquired close to the onset potential where the linear Tafel region began. Then, the curve comprising the overpotential *versus* the log of current density was constructed and the resulting slope gave the value of the Tafel slope according to the following equation:^1^*η* = *a* + *b* log *j*where *η* is the overpotential, *α* is the charge transfer coefficient (constant) and *b* is the Tafel slope.

#### Tafel slope calculations from EIS

Kinetically, OER is very sluggish due to the high solution resistance and charge transfer resistance. While calculating the Tafel slope to accurately study the intrinsic kinetics of the catalyst for OER, there should be 100% IR compensation. However, the above value is impossible for a catalyst producing high current densities and having solution resistance (*R*_s_) values greater than 6 Ω or a charge transfer resistance (*R*_ct_) value. Therefore, to precisely reflect the inherent kinetics of the active catalysts and to exclude the effect of high solution resistance here, we also calculated Tafel slopes for our best-performing catalysts (such as Ni-CNPs/FTO_500_) from impedance spectroscopy. The Nyquist plots were recorded at various applied potentials with an interval of 5 mV in between. In this way, 1/*R*_ct_ gives the exact value of the exchange current density at different overpotentials, and thus reflects the kinetics of the overall electrode process under a wide potential range.

#### Mass activity [MA (mA mg^−1^)]

MA was calculated using the following equation:^1^MA = *J*/(active mass of the catalyst)Here, *J* is the current density in mA cm^−2^ at a specific overpotential value of 0.35 V for comparing the electrocatalytic activities of all the thin film electrocatalysts.

#### Electrochemically active surface area (ECSA)

The electrochemically active surface area (ECSA) was calculated using CV mode by calculating the double-layer capacitance, considering the following relation:^1^ECSA = *C*_DL_/*C*_S_

First of all, the non-faradaic region (somewhere in between the oxygen and hydrogen region) in CV was identified by visual analysis of the cyclic voltammetry data, assuming that all the current in this potential range was due to the double-layer charging. Under this potential range, the CV was run at different scan rates (0.005, 0.01, 0.015, 0.02 V s^−1^). The charging current (*I*_c_) was calculated by identifying a middle potential range, and the ultimate current associated with this potential range was considered as the capacitive current or charging current. A plot of the scan rate *versus* the capacitive current was constructed, and the slope of this calibration curve gave the value of double-layer capacitance. Dividing C_DL_ with the *specific capacitance of the sample* (0.035 cm^−2^) gave the value of ECSA in cm^2^.

#### Determination of the surface concentration of Ni in Ni-CNPs/FTO from the CV curve

The charge passed value calculated by integrating the area (0.000115757) under the reduction peak from the potential *vs.* current curve was 0.00578787 Coulomb.Then, the no. of electrons = (0.00578787 C)/(1.602 × 10^−19^ C) = 3.612903 × 10^16^ electrons

The surface concentration of the Ni atom on the electrode was determined by dividing with the no. of electrons involved in the redox reaction, which is 1 for Ni^3+/2+^, therefore:Surface conc. of atoms = 3.612903 × 10^16^/1 = 3.612903 × 10^16^ atoms

Further details for the active concentration calculation for the other samples using a similar procedure are given in the ESI (Table S5†).

#### Determination of TOF from the integrated OER CV curve

We calculated the turnover frequency (TOF) at 1.58 V (*vs.* RHE) (*η* = 0.35 V) at a specific potential value. Furthermore, TOF was calculated at various applied potentials and the resulting TOF values are presented in Table S5.†

The turnover frequency was calculated at various potential values considering the following equation:
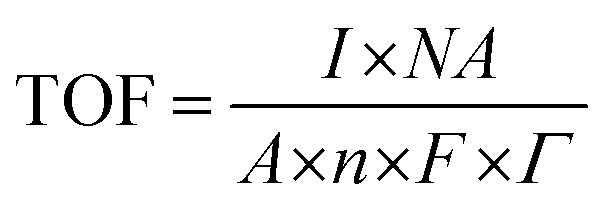
where, *I* = current value at a specified potential (*A*), *N*_A_ = Avogadro's constant, which is 6.022 × 10^23^, *A* = geometric area of the working electrode, which is 1 cm^2^, *N* = no. of electrons, *F* = Faraday constant, which is 96 485 Coulombs, *Γ* = surface concentration of atom on the electrode surface, TOF @ 1.58 V = [(0.00432) × (6.022 × 10^23^)]/[(1) × (4) × (96 485) × (0.00939× 10^19^)] = 0.18 s^−1^

Further details for the TOF calculation at different potentials are given in the ESI.†

#### Bulk electrolysis

Controlled-current electrolysis experiments were recorded, exploiting a similar setup as described above by applying the constant current of 2 mA cm^−2^ and 10 mA cm^−2^ for the fixed time interval of two hours, unless otherwise mentioned. The controlled potential electrolysis experiments were conducted applying the fixed potential value, and the change in the value of the current density was monitored as a function of time. For the CPE and CCE, the IR corrections were made manually. For all electrochemical measurements, the exposed area of the working electrode was *A* = 1 cm^2^.

## Results and discussion

The average nanoscale size and homogeneity of the Ni(OH)_2_ type colloidal nanoparticles were determined by a particle size analyzer. The analysis was carried out at room temperature.

**Fig. 1:**
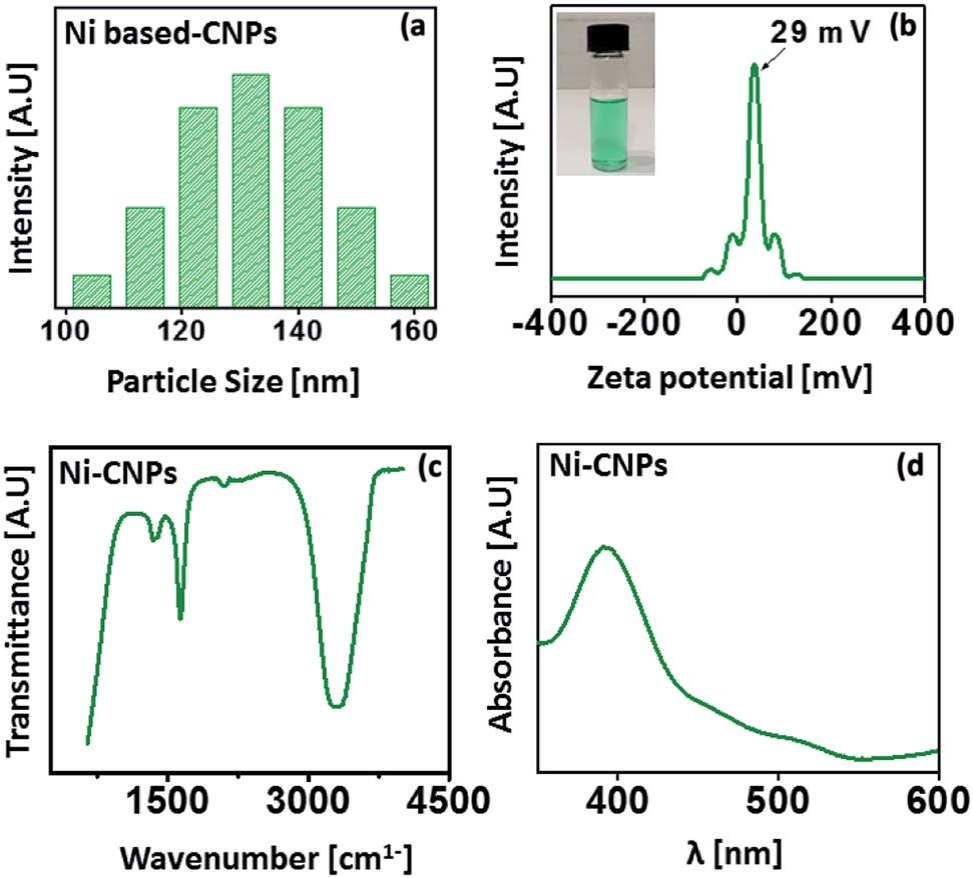
(a) Particle size distribution analysis; (b) zeta potential analysis; (c) FTIR spectrum and (d) UV-vis absorption spectrum for Ni-CNPs prepared in a borate buffer solution with pH ≈ 10.

The results reveal the average particle size, ranging from 100 nm to 150 nm for Ni-CNPs (Fig. 1a). For the sake of applicability, the nanoparticles must be stable and not accessible to agglomerations. The stability mainly depends on the electrostatic interactions between the positively charged metals and negative anions. The stability of Ni-CNPs was evaluated *via* zeta potential analysis. Ni-CNPs revealed a zeta potential of 29 mV, owing to their enhanced stability as presented in Fig. 1b. To sanction the formation of colloidal nanoparticles of the type M(OH)_2_ and/or M(OH)_2_/anions, FTIR spectroscopy was undertaken. The FTIR spectra for Ni-CNPs illustrate the appearance of an intense band in the vicinity of 3300 to 3600 cm^−1^, confirming the formation of the hydroxide of that metal (Fig. 1c). Furthermore, the UV-vis absorption spectrum for Ni(OH)_2_ reflects the appearance of an intense peak at about 360 nm, which could involve some electronic transition from the α-Ni(OH)_2_-type active phase (Fig. 1d).^37^ Thus, the initial analysis illustrates the formation of nano-colloids of the metal-hydroxide type. To study the effect of the pH value of the borate buffer, Ni-colloids were also prepared at varying pH values of the borate buffer system from pH = 10 to 12. Scanning electron microscopic (SEM) images of the Ni-colloids prepared in a borate buffer of pH ≈ 10 showed nanoscale features, whereas those obtained in a borate buffer system having pH ≈ 11 and pH ≈ 12 showed microstructures grown on FTO having a relatively larger particle size (Fig. S1–S3†). This might be due to agglomeration and suspension formation that occurred at the higher pH value.^7,31^ Therefore, pH ≈ 10 is considered an optimum pH for nano-colloidal formation.

**Fig. 2:**
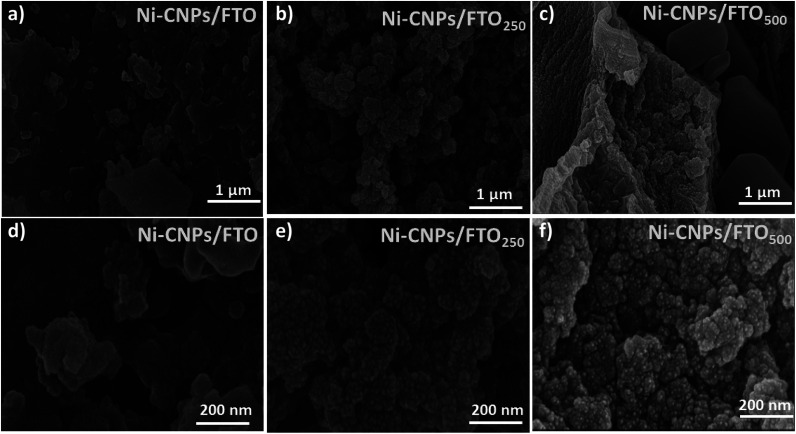
Scanning electron micrograph images for Ni-CNPs/FTO: (a) low magnification image and (d) high magnification image; Ni-CNPs/FTO_250_ (b) low magnification image and (e) high magnification image; Ni-CNPs/FTO_500_ (c) low magnification image and (f) high magnification image.

For the preliminary catalytic study, the Ni-CNPs were deposited onto FTO substrates in the form of a thin-film. Primarily, the physical and physicochemical attributes of the Ni-CNP-derived thin film nano-electrocatalyst were evaluated by various analytical approaches. The surface morphology of the thin film nano-electrocatalysts was examined *via* scanning electron microscopy. The SEM micrograph for the Ni-CNPs prepared at pH ≈ 10 indicates the non-homogeneous film comprising micro-aggregates of flake-like structures grown in an irregular fashion on the electrode surface (Fig. 2a). The enlarged SEM view (Fig. 2d and S1†) reveals that a uniform layer of nanoparticles covered each micro-flake-like structure with definite spaces in between, thus indicating micro-nano porosity. The estimated size of the nanoparticles is in the range of 20–30 nm.

Furthermore, by applying the post-heat treatments to the catalytic film, the structural and morphological modifications were observed and also revealed by SEM images. The scanning electron micrograph of an annealed film of Ni-CNPs at 250 °C (Ni-CNPs/FTO_250_) shows a galaxy-type accumulation of fine NPs evolving from the base and covering the entire surface of the electrode (Fig. 2b). A magnified view reveals spherical NPs that are grouped together to form small clumps, which are linked with each other in a coral-reef type alliance (Fig. 2e and S4†). The film looks non-homogeneous in thickness with certain spaces in-between the aggregates of the Ni-based CNPs. The metal NP size is about 15 nm, having a narrow size distribution. The presence of mesopores between the adjacent particles can also be observed throughout the entire volume of the catalytic film. The SEM image of the Ni-CNPs annealed at 500 °C (Ni-CNPs/FTO_500_) shows the distorted micro-globular features containing NPs spread in a compact and irregular fashion on the electrode surface (Fig. 2c). The enlarged view reveals the nanoscale features of the catalytic film. The nanoparticles are spherical aggregates and dispersed on the electrode surface, with a size in the range of 10 nm (Fig. 2f and S5†). From the comparative SEM analysis, it can be seen that all three Ni-CNP-sample-derived thin-films are nanostructured materials. Much smaller nanoparticles with nanometer-size distribution can be obtained by applying the annealing operations to the catalytic film. However, the SEM image for the simple FTO without the catalyst coatings did not show any nanoscale structural or morphological attributes (Fig. S6†).

Elemental bulk compositional analysis of these nano-electrocatalysts derived from colloidal Ni-NPs before and after annealing was conducted *via* SEM-EDS (energy dispersive X-ray) measurements. The EDS spectra of all the electrocatalytic thin-films (on FTO glass substrates) are presented in Fig. S7 to S9.† The presence of various effective metal constituents in the thin-film anode materials is shown in Table S2.† From the analysis, it is observed that the oxygen content of the thin film is high in both annealed and unannealed samples. The EDS spectra of the nickel-based thin-film nano-electrocatalysts show no significant signatures for boron. This might be due to the insufficient amount of boron incorporated in the catalytic layer, which is below the detection limit of the instrument. Minute signatures of Si are observed in the samples, which originated from the FTO-coated glass substrates.

**Fig. 3:**
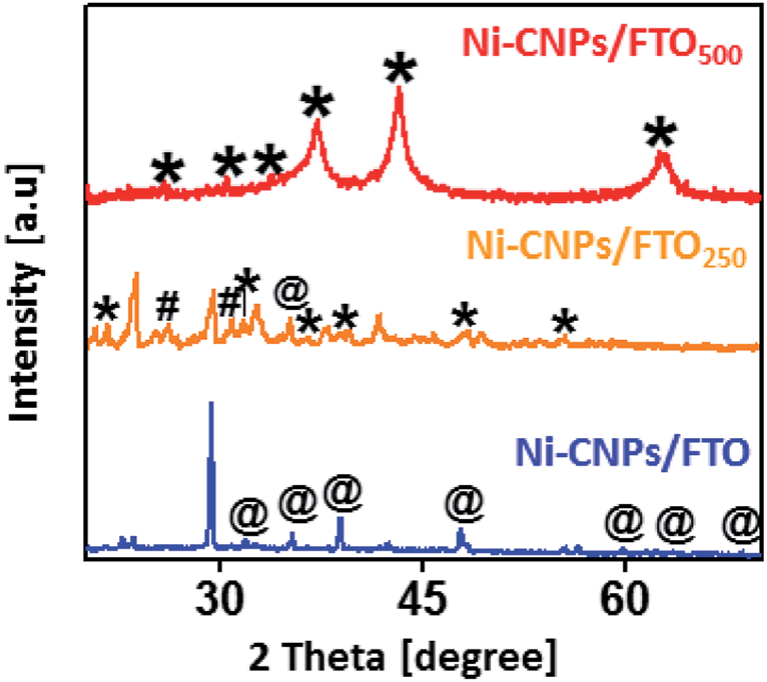
X-ray diffraction pattern analysis for Ni-CNPs/FTO, Ni-CNPs/FTO_250_ and Ni-CNPs/FTO_500_ type nano-electrocatalysts derived from the Ni-CNPs where the symbols *, # and @ in the figure represents the Ni_2_O_2_OH, NiO_2_ and Ni(OH)_2_ phases, respectively.

The active phase of the thin-film nano-electrocatalysts was identified by powder X-ray diffraction measurements. The XRD spectra for Ni-CNPs/FTO and Ni-CNPs/FTO_500_ show high-intensity diffraction peaks for the Ni(OH)_2_ phase^37^ and Ni_2_O_2_OH phase,^38^ respectively. In contrast, the diffraction pattern for Ni-CNPs/FTO_250_ shows the presence of a mixture of Ni_2_O_2_OH^38^ and NiO_2_,^39^ and a single diffraction peak contribution for the Ni(OH)_2_ phase was also observed as shown in Fig. 3. In the X-ray diffraction pattern for all catalysts, the appearance of intensive and sharp peaks illustrates the growth of crystal-type mixed structures on the conductor surface, and thus confirms the crystalline nature of the nano-electrocatalysts. To further confirm the presence of metal hydroxides and oxides with varying oxidation states on the catalytic surface, Raman spectroscopy was used. Fig. S10† shows the Raman spectra for the nickel-based nano-electrocatalyst discussed in this study. The Raman spectra for Ni-CNPs/FTO indicated the appearance of discrete bands at 500–600, 711, 989 and 1045 cm^−1^ that are assignable to Ni(OH)_*x*_, as described previously.^40^ The Raman spectra for Ni-CNPs/FTO_250_ and Ni-CNPs/FTO_500_ show obvious bands in the range of 400 to 600 cm^−1^ and at 740 and 1020 cm^−1^, which are indicative of NiO nanoparticles.^41^ Therefore, it can be deduced from these results that all of the colloidal NP-derived thin-film nano-electrocatalysts discussed here are composed of mixtures of various metal-hydroxide/oxide phases. It was also observed that the dominating phase in the un-annealed sample is M(OH)_*x*_, whereas the annealed samples demonstrated the presence of numerous phases of metal-oxides with different oxidation states in the catalytic film.^42^

**Fig. 4:**
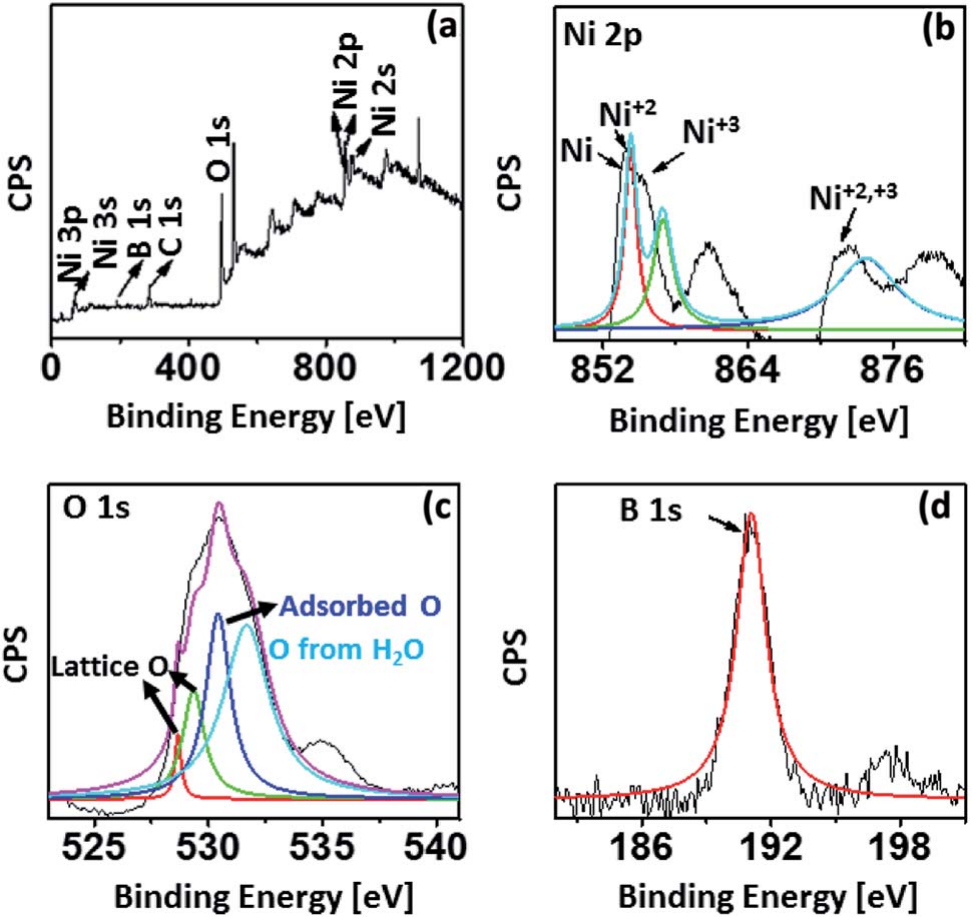
XPS spectrum for the Ni-CNPs/FTO_500_-type nano-electrocatalyst, showing the (A) survey spectrum; high-resolution XPS for (B) Ni; (C) O and for (D) B.

Furthermore, to exactly determine the possible oxidation states, chemical and elemental composition of the nano-catalysts, X-ray photoelectron spectroscopy (XPS) analysis was undertaken for Ni-CNPs/FTO_500_ (based on the electrochemical data as discussed in a later section, and the formation of more fine nanoparticles as presented *via* SEM analysis). The XPS analysis for Ni-CNPs/FTO_500_ is shown in Fig. 4a–d. The survey and high-resolution deconvoluted core spectra for Ni 2p illustrate the presence of intense peaks at 855.1 eV (Ni 2p_3/2_) and 873.1 eV (Ni 2p_1/2_) for Ni^+2^.

Ultimately, the Ni^+2^ and Ni^+3^ phases were shown to coexist in the catalysts, along with C (oxygenated and free carbon) (Fig. S11†), O and B as the main elemental constituents.^43^ Distinct shoulder peaks at about 852 eV (Ni 2p_3/2_) and 872 eV (Ni 2p_1/2_) demonstrate the presence of elemental nickel, which may form due to the surface reduction of some of the nickel oxide.^43*a*^ More interestingly, B 1s is also shown to be incorporated in the catalyst from the borate buffer through the XPS results, which was not revealed earlier by EDS analysis (Fig. 4d).^43*b*^ Conclusively, the surface and bulk compositional analyses reveal the presence of mixed structures with various oxidation states in the catalyst surface. More advantageously, the pronounced signatures for carbon and boron indicate that these species can be easily incorporated in the catalyst layer by following the much simpler colloidal route. The nano-scale morphological features of all electrocatalysts let us comparatively evaluate the electrochemical activity of all annealed and un-annealed Ni–CNP-derived catalysts under somewhat similar electrochemical conditions. The kinetic parameters that were selected to benchmark the catalyst performance include: (1) the overpotential, where the linear Tafel region begins, or more specifically, is called the onset overpotential; (2) the overpotential needed to achieve a current density of 10 mA cm^−2^, which is merited for the 10% efficient conversion of solar energy into fuels; (3) mass activity and TOF of the catalyst at an overpotential of 0.35 V; (4) Tafel slope (mV dec^−1^); (5) exchange current density calculated from EIS; (6) short term stability of the catalyst during the controlled potential water electrolysis for 2 hours applying constant potential; and (7) the short-term stability of the catalyst during the controlled-current water electrolysis at the constant current density value of 2 mA cm^−2^ and 10 mA cm^−2^.

**Fig. 5:**
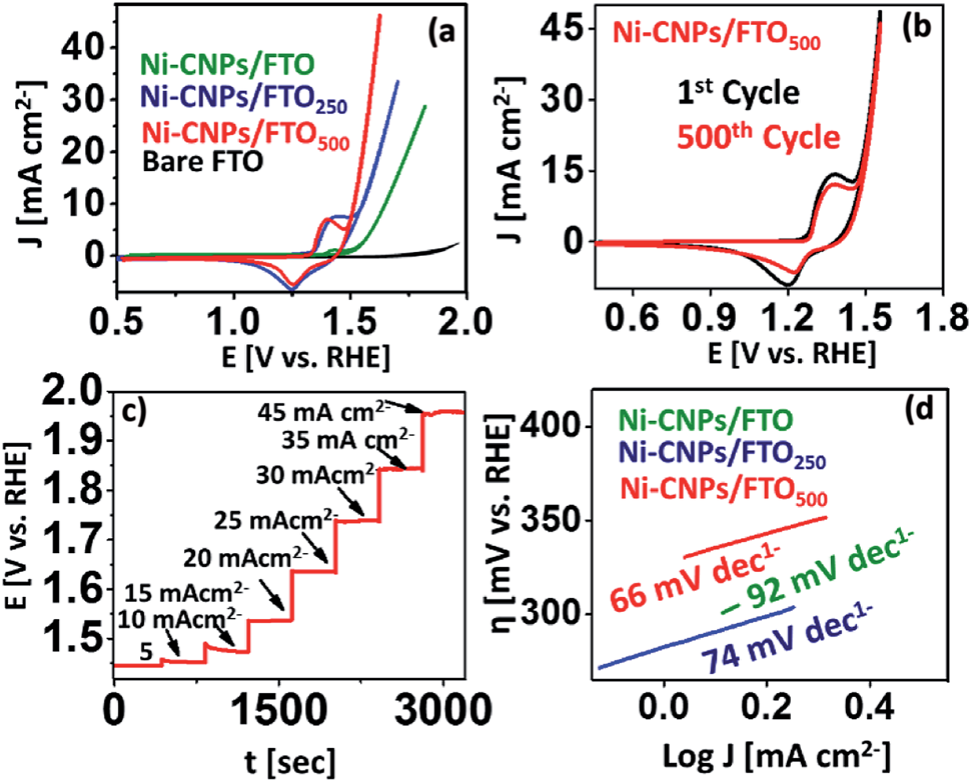
Electrocatalysis; representative cyclic polarization curves of OER at a scan rate of 5 mV s^−1^ for (a) (green) Ni-CNPs/FTO, (blue) Ni-CNPs/FTO_250_, and (red) Ni-CNPs/FTO_500_; (b) a representative multiple scan cyclic voltammogram for Ni-CNPs/FTO_500_ (black), 1st cycle (red), and 500^th^ cycle in 0.1 M aq. NaOH electrolyte solution at a scan rate of 50 mV s^−1^; (c) multistep CCE presenting the potential profile for the consecutive current density increments from 5 mA cm^−2^ to 45 mA cm^−2^ for Ni-CNPs/FTO_500_; (d) Tafel plot (overpotential *versus* log of current density curve) calculated from the polarization curve for the Ni-CNP-derived nano-electrocatalysts in an aq. NaOH electrolyte solution having a pH ≈ 13.

The water oxidation catalysis on Ni-CNPs/FTO, Ni-CNPs/FTO_250_, and Ni-CNPs/FTO_500_ are primarily scrutinized by cyclic voltammetry in a 0.1 M aq. NaOH electrolyte solution with pH ≈ 13. Fig. 5a shows the steady-state polarization curve for the un-annealed and annealed Ni-CNP-based electrocatalysts. The anodic current for nickel oxidation starts growing sharply at ∼1.40 V (*vs.* RHE). The oxidative current is indicative of the oxidation of the Ni species to a higher status as observed previously, leading to the generation of more active catalytic nanostructures.^25^ Following this, the onset current for the water oxidation reaction is 1.55 V (*vs.* RHE), which is lower than the previously reported Ni-based system.^42^ A current density of 10 mA cm^−2^ was achieved at a mere 1.65 V (*vs.* RHE), reaching >28 mA cm^−2^ at 1.82 V (*vs.* RHE). The CV profile for the Ni-CNPs/FTO_250_ electrocatalytic sample shows the oxidative prefectures at about 1.3 V (*vs.* RHE), which increases in magnitude and after attaining maximum height, is quickly followed by a catalytic wave at a mere potential of 1.53 V (*vs.* RHE). The potential is more negative for the catalytic peak relative to the un-annealed Ni-CNPs/FTO sample. The onset overpotential is calculated to be 300 mV. The catalytic wave increases sharply and reaches a current density of 10 mA cm^−2^ at just under the potential of 1.55 V (*vs.* RHE) [*η* = 320 mV], and is quickly accompanied by the generation of tiny oxygen bubbles at the electrode that could be clearly seen coming out of the FTO surface during the oxidative scan. The oxygen evolution current density rises with further increases in the potential, and attains a current density of >33 mA cm^−2^ at 1.71 V (*vs.* RHE), showing the remarkable electrocatalytic activity of the Ni-CNPs/FTO_250_-type electrocatalyst for water oxidation. The polarization curve for Ni-CNPs/FTO_500_ presents the appearance of an oxidative peak at 1.38 V (*vs.* RHE), which after attaining a maximum height at 1.44 V (*vs.* RHE), undergoes a slight decline and is quickly followed by a sharp catalytic peak. The catalytic wave for the onset of water oxidation for Ni-CNPs/FTO_500_ appeared at about 1.48 V (*vs.* RHE). A current density of 10 mA cm^−2^ which is mandatory for the 10% efficiency of the solar to fuel conversion systems,^42^ was achieved at 1.55 V (*vs.* RHE) [*η* = 320 mV]. A peak current density of >40 mA cm^−2^ was ultimately observed at the potential of 1.71 V (*vs.* RHE). It is notable that for Ni-CNPs/FTO_500_ following Ni oxidation, the realization of such a high current density within the narrow potential regime so close to the oxidative pre-features of Ni demonstrates the faster kinetics and much enhanced electrocatalytic efficacy of the material under study. Such behavior supports the faster electronic transfer and efficient O–O bond formation on the easily obtainable ultrafine nano-surfaces.^43^

In the oxidative scans of all the nickel-based materials discussed here, the appearance of the small redox couple at about 1.2 to 1.45 V (*vs.* RHE) can be assigned to the redox reaction of the Ni^2+^/Ni^3+^ species (Fig. S12†). These features were also observed in many other nickel-based electrocatalytic assemblies reported earlier.^31,42^ The appearance of this oxidative pre-featured in all nickel-based catalytic materials is indicative of the presence of a high valence nickel species as the active material that favors water oxidation catalysis.^42^ This is ascribed to the formation of the finer nanoscale catalytic CNPs on the electrode surface as revealed by SEM microscopy (Fig. 2). However, by further increasing the annealing temperature up to 750 °C, the catalyst showed substantial activity and high current density, although the onset potential was high as revealed in Fig. S13.† Therefore, further increasing the annealing temperature for the Ni-based materials was not undertaken, and 500 °C was considered the optimal temperature for generating the high surface area nanoscale catalytic materials. Overwhelmingly, it was gathered that the Ni-CNP-derived electrocatalyst are electrochemically active and can be used directly as an anode material for water oxidation catalysis. Furthermore, the post-annealing at 250 °C or 500 °C can further enhance the morphological features, such as the roughness and surface area of the film. The thus-obtained film materials are highly reliable for long-term applications as well. Based on the preliminary CV data, we considered Ni-CNPs/FTO_500_ the best performing electrocatalyst among the other series of nickel materials discussed in this study (Table S3†). The lowest onset overpotential for them is attributed to the formation of the more intense nanopattern oxides, as revealed by SEM microscopy.

Further, the effect of various substrates on the electroactivity of the Ni-CNPs is also evaluated. In this quest, the Ni-CNPs were deposited onto nickel foam (NF) and carbon cloth (CC) surfaces using a similar method of electrode preparation, as described above. However, catalyst loading was increased from 80 to 100 μL due to the high surface area and porosity of these substrates.^44^ The electrocatalytic activity of the thus-obtained electrodes was tested under the employed electrochemical conditions. Previously, it was shown that the Ni–Co–Ni oxide produced 10 mA cm^−2^ at 240 mV on the (glassy carbon) GC and the overpotential was reduced to 203 mV when the catalysts were supported on the highly conductive nickel foam substrate.^44^ Cyclic voltammetry and impedance results reveal that the catalysts coated on highly conductive NF and CC substrates revealed much more efficient electrocatalytic activity and lower charge transfer resistance towards OER as presented in Fig. S14 and S15† (comparative results are presented in Table S4†). The very impressive OER performance of the Ni-CNPs coated on NF and CC is attributed to the high intrinsic activities of these materials and fabulous structural properties, such as the porous surface (Fig. S16†). The presence of a large number of electroactive sites on NF and CC that are in contact with the electrocatalysts can further enhance multiple electron transfer processes, and can make the overall process energy efficient.^44,45*c*^ Also, the bare NF and bare CC present a much lower charge transfer resistance relative to bare FTO when the impedance is conducted in 0.1 M aq. NaOH solution at an applied potential of 450 mV (Fig. S16†). In spite of the much improved catalytic performance of the easily obtainable Ni-CNPs on NF and CC relative to the FTO electrodes, the transparent conductive substrates (such as FTO) are intentionally employed here to further study the true intrinsic properties (*e.g.*, electroactivity, kinetics, and stability) of the electrocatalyst merely towards OER. As for the successful designing and engineering of the electrocatalyst, a critical evaluation of its exclusive response towards the desired process is highly required. Therefore, further catalytic studies are conducted on the inert FTO substrate as it is oxidatively stable, tolerant of mechanical abrasion and shows only the catalytic efficacy of electrocatalysts without any interference from the substrate.^42,45*b*^

The durability of the aforementioned best-performing catalysts was evaluated *via* accelerated degradation testing through a series of multiple-scan cyclic voltammetry experiments at a higher scan rate of 50 mV s^−1^ (Fig. 5b). It is notable that the electrochemical activity of Ni-CNPs/FTO_500_ is very stable for up to 500 consecutive CV cycles without any observable degradation. For every scan, the potential for the peak oxidative current density of >45 mA cm^−2^ was 1.71 V (*vs.* RHE), which is ascribed to the remarkable stability of the catalyst under harsh oxidative conditions and presenting a consistent electrochemical behavior for water oxidation catalysis.

In addition, consistency in the catalytic performance of Ni-CNPs/FTO_500_ for the water oxidation reaction (WOR) was also evaluated *via* a series of multistep-controlled current electrolysis and controlled potential electrolysis experiments, as illustrated in Fig. 5c and S17.† In pursuit of this, the catalyst was deliberately held at a constant current density for 400 seconds and an increase in the potential was studied as a function of time. It was observed that, by increasing the applied current density under a controlled experiment, the potential value also rose accordingly and was maintained for 400 s, thus showing consistency in its performance for OER using Ni-CNPs/FTO_500_. In another experiment, the catalyst was held at the constant potential value for a time interval of 300 seconds, and a change in the value of the current density was observed as a function of time. Upon increasing the value of the applied potential, the ultimate current density increased and was soon stabilized as presented in Fig. S17.† This feature thereby illustrates the fine consistency in the catalytic performance for OER under optimized electrochemical conditions, which is required for the long-term application of any catalytic system.

### Electrochemical kinetics

The CV data reveal that all nanoscale catalysts discussed in this study are electrochemically active for oxygen evolution. For comparative analysis, the widely used kinetic parameters (such as mass activity, exchange current density, TOF, electroactive accessibility, ECSA calculations, and Tafel analysis) were conducted for linking the electrocatalytic efficiency of various catalytic materials for oxidizing the water under similar employed conditions. In pursuit of this, the Tafel slopes were calculated from the polarization curve considering the linear region of the CV scan, and the resulting *η versus* the log of *J* curves for the Ni-based catalytic systems are illustrated in Fig. 5d. Tafel slope values of 92 mV dec^−1^, 74 mV dec^−1^ and 66 mV dec^−1^ were observed for Ni-CNPs/FTO, Ni-CNPs/FTO_250_ and Ni-CNPs/FTO_500_, respectively (Table S5†).

**Fig. 6:**
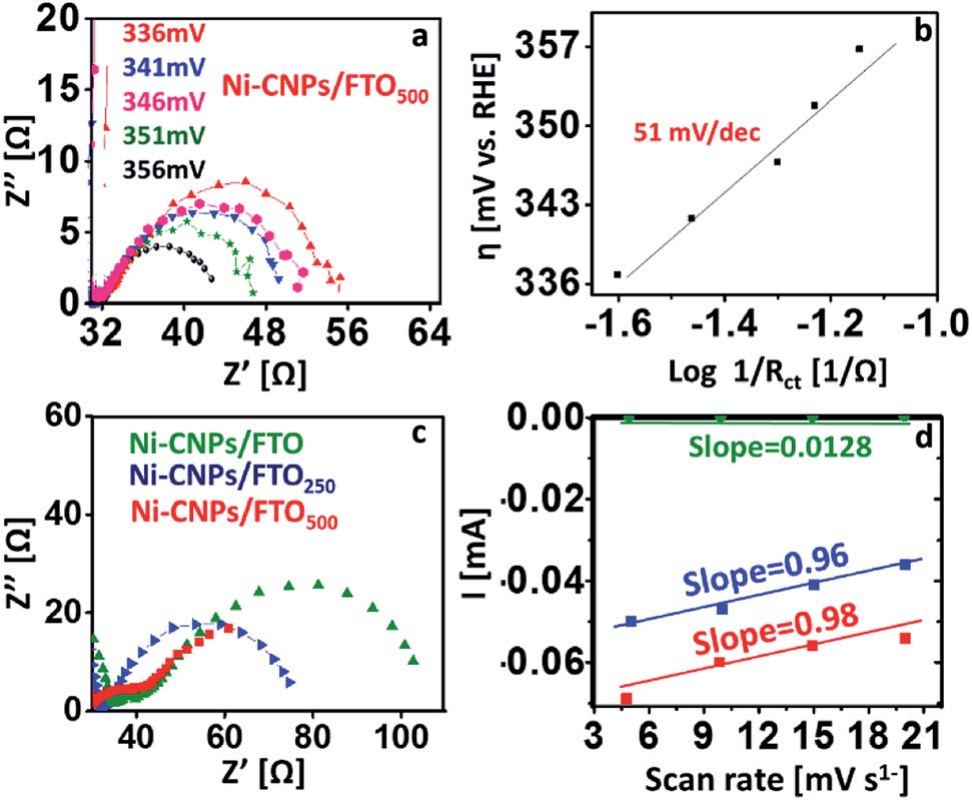
Electrocatalysis; (a and b) electrochemical impedance spectroscopy for calculating the Tafel slopes, presenting the Nyquist plots at various potentials with an incremental increase of 5 mV. The corresponding Tafel plots show the applied potential *vs.* inverse *R*_ct_ on a logarithmic scale for Ni-CNPs/FTO_500_; (c) representative Nyquist plots for the Ni-based nano-electrocatalysts at the applied potential of 1.58 V *vs.* RHE; (d) scan rate *versus* current curves, and the slope of which demonstrates the double-layer capacitance values for the Ni-CNP-derived catalytic systems in 0.1 M aq. NaOH solution.

Perhaps the discussed electrocatalysts revealed slightly higher Tafel slope values that might have originated from the high resistance of the electrochemical system. Therefore, to reveal the real catalytic aptitude of the electrocatalyst and to avoid the influence of the series resistance on the calculated Tafel slopes, the Tafel slope for best-performing catalysts (such as Ni-CNPs/FTO_500_) is also calculated here *via* EIS measurements.^44^ The Nyquist plots were measured at various applied potentials with the interval of 5 mV in between measurements, and a logarithmic reciprocal of *R*_ct_ was plotted against the overpotential where the slope gave a true value of the Tafel slope at 51 mV dec^−1^ for Ni-CNPs/FTO_500_. This truly reflects the inherent kinetics of the catalyst without the influential effects of the series resistance as shown in Fig. 6a and b. A smaller Tafel slope is compulsory as the electrocatalyst is desired to operate over a narrow potential window, while producing a high value of current density.^45*a*^ The smaller values of the Tafel slopes also reflect the resistance-free nature of the catalysts for OER catalysis. Furthermore, electrochemical impedance spectroscopy is used to gain deep insight into the catalyst kinetics in terms of the charge transfer resistance at the so-called electrode/electrocatalyst-electrolyte interphase under the employed electrochemical conditions. The corresponding Nyquist plots for the Ni-based materials are shown in Fig. 6c.

To precisely calculate the *R*_ct_ values, the simplified Randles circuit was fitted using Nova software and the resulting values are presented in Table S5.† From the Nyquist plots, the electrochemical kinetics of the reaction is directly linked with a diameter of a semicircle; a smaller diameter is indicative of faster kinetics.^45*b*^ It is evident from the results that smaller values of *R*_ct_ are observed (calculated from the low-frequency region of the semicircle) for Ni-CNPs/FTO_500_ (18 Ω), demonstrating its intrinsic catalytic activity to reduce the resistance of the system and thus making the sluggish OER feasible under the employed electrochemical conditions, which is obviously desirable. Furthermore, the smaller *R*_ct_ also demonstrates that the catalyst is primarily catalyzing only OER, rather than any other residual reaction that may or may not be taking place within the electrochemical system. The electrochemically active surface area was calculated *via* double-layer capacitance measurements (Fig. 6d), and a high surface area of 27.2 cm^2^ was observed for the Ni-CNPs/FTO_500_-type catalytic systems. The ECSA values of all catalytic systems discussed in this study are presented in Table S5 and Fig. S18–20.†

**Fig. 7:**
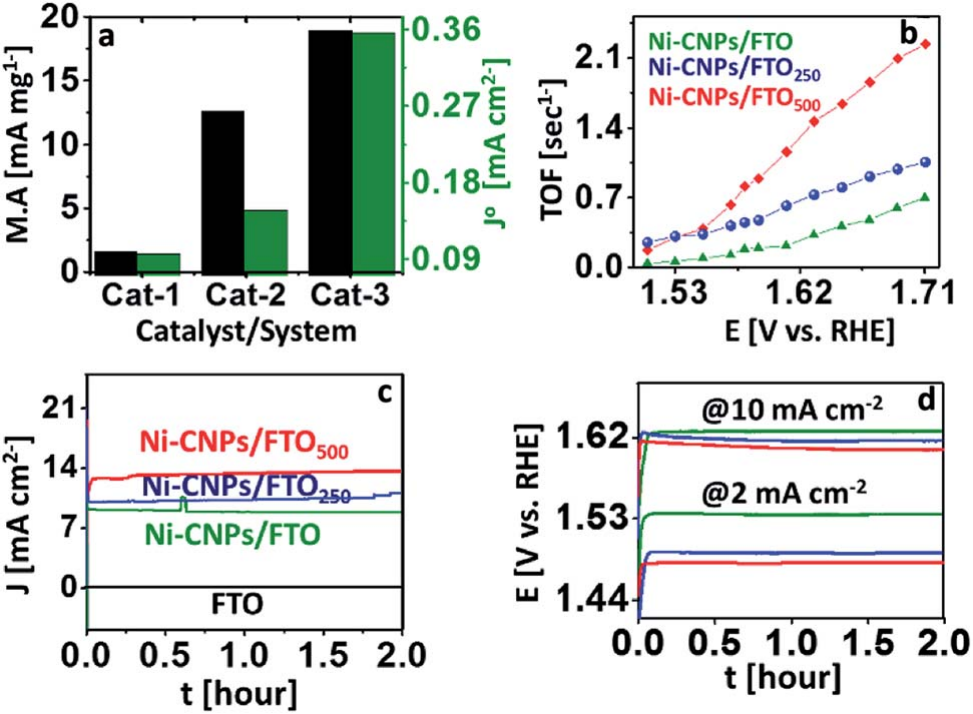
Electrocatalysis; (a) mass activity at 1.58 V (*vs.* RHE) and exchange current density demonstration for (Cat-1) Ni-CNPs/FTO, (Cat-2) Ni-CNPs/FTO_250_, and (Cat-3) Ni-CNPs/FTO_500_; (b) TOF calculations for the Ni-based nano-electrocatalysts at various applied potentials; (c) time *versus* current density curves for continuous 2 hours for (green) Ni-CNPs/FTO @ 1.65 V (*vs.* RHE), (blue) Ni-CNPs/FTO_250_ @ 1.55 V (*vs.* RHE), and (red) Ni-CNPs/FTO_500_ @ 1.55 V (*vs.* RHE); (d) time *versus* potential V (*vs.* RHE) curves at a fixed current density of 2 and 10 mA cm^−2^ for the Ni-based nano-electrocatalysts, (green) Ni-CNPs/FTO, (blue) Ni-CNPs/FTO_250_, and (red) Ni-CNPs/FTO_500_ in 0.1 M aq. NaOH electrolyte solution.

The mass activity of all nanoscale catalysts was calculated at a fixed overpotential value. It may refer to the current density per active mass of catalysts or the rate of oxygen evolved per active mass of catalysts per time.^42^ Here, as a descriptive exercise, the mass activity was calculated by dividing the value of the current density per geometric area achieved at an overpotential of 0.35 V to the active mass of the catalyst (details are described in Experimental section ESI†). The method employed was chosen because it comprises the easily measured parameters. The corresponding mass activity of each catalyst is shown in Table S5.† The choice of *η* = 0.35 V is based on the assumption that the 10% efficient solar water splitting devices should operate at 10 mA cm^−2^ with a maximum of 0.35 V overpotential for the oxygen evolution reaction.^1^ From Fig. 7a, it is clear that the higher value of the mass activity was observed for Ni-CNPs/FTO_500_ (56.33 mA mg^−1^ @ 0.35 V) among other candidates, which is consistent with the CV data and shows the substantial activity of the catalysts. Furthermore, to precisely evaluate and compare the intrinsic activity of all catalysts for catalyzing the OER reaction, we calculated the exchange current density (*J*°) by taking the charge transfer resistance into account. The highest value of (J°) is observed for Ni-CNPs/FTO_500_ (0.35 mA cm^−2^) due to the smaller observed *R*_ct_ values for these catalysts relative to other materials discussed in this study (Fig. 7a). The *J*° values for all catalysts are shown in Table S5.†

For a better understanding of the kinetics of all nano-electrocatalysts, we calculated TOF by determining the electroactive sites constituting Ni on the catalytic interface, assuming all atoms are catalytically active. In this quest, the area under the reduction peak of the Ni redox couple directly measured from polarization curves was integrated, considering the optimal potential range as shown in Fig. S21.† Consequently, the higher TOF value is observed for Ni-CNPs/FTO_500_ (0.79 s^−1^ @ 0.35 V) relative to other catalytic candidates discussed here (Table S5†). Furthermore, we calculated TOF for all catalysts at various applied potentials, considering the corresponding values of the current densities (Fig. 7b and Table S6†). It must be noted that TOF can only be used as an approximate guide to comparatively study the catalytic activity of the different materials employed under somewhat similar electrochemical conditions. The electrochemical activity of our best-performing catalysts (such as Ni-CNPs/FTO_500_) is also compared with the previously reported Ni-Based catalytic systems in Table 1. It is seen that Ni-CNPs/FTO_500_ initiates OER at the relatively lowest onset overpotential and the current density is also lower. An impressive Tafel slope of 51 mV dec^−1^ demonstrates the excellent kinetics of OER on the electrode.

**Table 1:** Comparative electrochemical activity of our best-performing catalyst with other systems reported earlier^a^

Catalyst/system	Electrolyte	(*η*) @ onset (mV)	η (mV) at 10 (mA cm^−2^)	Tafel slope (mV dec^−1^)	Ref.
Ni-CNPs/FTO_500_	0.1 M NaOH	250	320	51	TW
Ni/N-graphene	0.1 M KOH	—	430	188.6	46
NiCo/N-graphene	0.1 M KOH	350	—	614	47
NiO_*x*350_ @ FTO	0.1 M NaOH	320	610	147	42
NNCNTAs	0.1 M KOH	310	460	65	48

a TW = this work; Ni/N-graphene = nitrogen-doped graphene film-confined nickel nanoparticles; NiCo/N-graphene = nitrogen-doped graphene hydrogel/NiCo double hydroxide; NNCNTAs = three-dimensional Ni@NiCo hydroxide nanotube arrays.

### Short-term stability testing

The electrochemical durability during the vigorous oxidative reaction is a decisive factor that determines the industrial implication of a catalytic system. Owing to the remarkable electrochemical activity of all the catalytic materials discussed here, we further evaluated the short-term stability of the N-CNP-derived nano-electrocatalysts *via* a series of controlled-potential electrolysis and controlled-current electrolysis experiments for two hours.

Fig. 7c demonstrates that all the Ni-CNP-derived annealed and un-annealed nano-sized catalytic systems present constant values of current densities during the CPE experiments for at least two hours of electrolysis without showing any degradation or decrease in catalytic performance. However, an increased value of current density is observed for Ni-CNPs/FTO_250_ and Ni-CNPs/FTO_500_, confirming the electroactive nature of the system. Likewise, the controlled current electrolysis experiments at a fixed current density of 2 and 10 mA cm^−2^ for two hours also confirmed the stable and electroactive nature for all catalytic systems (Fig. 7d)^45*c*^ (Table S7†).

### Extended stability

The remarkable electrocatalytic activities of all Ni-based thin-film nano-electrocatalysts let us evaluate the extended stability of all catalysts under harsh oxidative conditions. For the long-term stability testing, the catalytic performance of the Ni-CNPs/FTO, Ni-CNPs/FTO_250_ and Ni-CNPs/FTO_500_-type electrocatalysts were evaluated using CPE experiments in 0.1 M aq. NaOH electrolyte solution (pH ≈ 13).

**Fig. 8:**
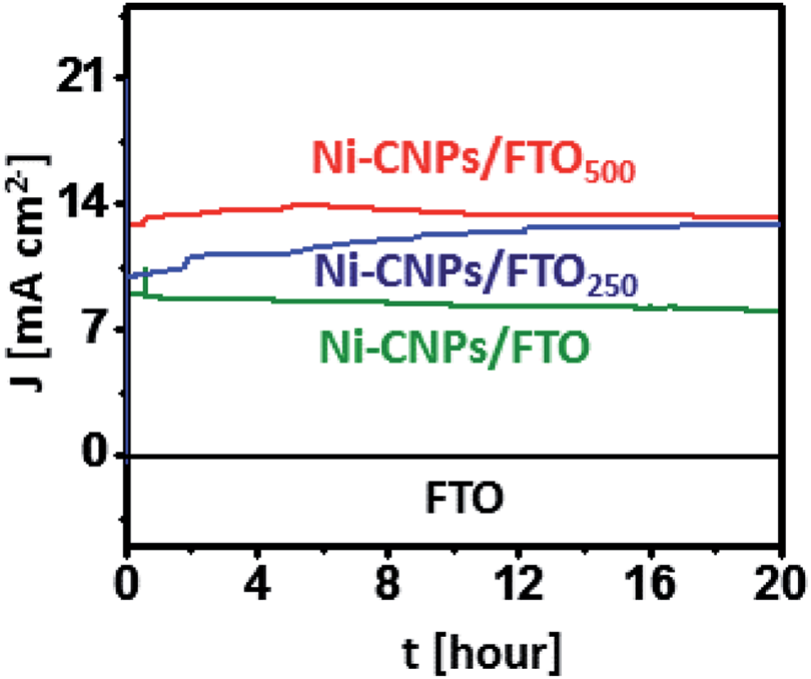
Extended period anodic water oxidation test during controlled potential bulk electrolysis using (a) (green) Ni-CNPs FTO @ 1.65 V (*vs.* RHE), (blue) Ni-CNPs/FTO_250_ @ 1.55 V (*vs.* RHE), and (red) Ni-CNPs/FTO_500_ @ 1.55 V (*vs.* RHE) in 0.1 M aq. NaOH electrolyte (pH = 13). [Black represents the bare FTO electrode without catalyst loading @1.65 V (*vs.* RHE)].

Fig. 8 shows that Ni-CNPs/FTO produced an excellent current density of 8.9 mA cm^−2^ at a fixed potential of 1.65 V (*vs.* RHE) [*η* = 420 mV], which remained stable and achieved a value of 8.3 mA cm^−2^ after 20 hours of continuous electrolysis with only 10% degradation. The catalyst Ni-CNPs/FTO_250_ produces an excellent current density value of 9.8 mA cm^−2^ at *t* = 0, which increases and attains a value of 12.8 mA cm^−2^ at *t* = 20 hours @1.55 V (*vs.* RHE) [*η* = 320 mV]. This feature proved that the catalyst under study is an electroactive species and is highly applicable for long-term applications. Finally, for comparative analysis, the electrochemical durability of Ni-CNPs/FTO_500_ was evaluated under the same electrochemical conditions as described above by holding the catalyst at a fixed potential of 1.55 V (*vs.* RHE) (*η* = 320 mV). It can be seen that the catalyst is highly active and producing a continuously increasing value of current density from 12 mA cm^−2^ at *t* = 0 to >13 mA cm^−2^ at *t* = 20 hours (Table S8†).

This thin-film electrocatalyst also exhibits some electroactive content, which is capable of activation under the harsh oxidation conditions with the passage of time.^42^ This feature proves that the Ni-based nano-pattern electrocatalyst presented here and prepared by a very simple, time-effective and straightforward colloidal approach are superior electroactive materials, and the electrocatalytic durability can be further enhanced by applying post-heat treatments in the low-temperature range for a short time span.

### Post OER-catalysis material characterizations

Furthermore, to know about the changes in the chemical structure of the electrocatalyst after long-term catalytic operations, XPS analysis was used (Fig. S22†). The XPS spectrum for Ni-CNPs/FTO_500_ was taken after CPE analysis was conducted for 20 hours. It was observed that the Ni 2p spectra showed a marked increase in the intensity of the peaks. The Ni^3+^ species is evidenced by the presence of a peak at 857.2 eV assigned to α-NiOOH, which is unstable and readily converted to the Ni(OH)_2_-type structure. The XPS spectrum of oxygen acquired after CPE reveals that more oxygen was adsorbed onto the catalyst substrate due to the formation of nickel hydronium oxide, which is believed to be particularly participating in the enhancement of the catalytic activity of the material. The most intense peak at 531.5 eV is due to the surface hydroxyl group. The distinct peak at 530 eV is attributed to Ni(OH)_2_, which is typically consistent with lattice oxygen. The remaining peak at 532 eV corresponds to adsorbed water. Therefore, it is reasonable to conclude that during anodic catalysis, there is the surface formation of α-NiOOH, which is an active catalytic site.^7*b*^

## Conclusions

Transition metal oxides/hydroxides-based nanoscale electrocatalysts are very promising for catalyzing OER due to the high redox potential, low price and abundance. However, the facile fabrication methods and hand-on exploitation of nanoparticle-based electrocatalytic assemblages are highly limited due to expensive chemical setups, lengthy preparative protocols, and the complex functionalization reaction. Furthermore, agglomeration, sintering or dissolution of nanoparticles during the catalysis reaction is also an issue. Therefore, the development of some straightforward methods using easily available materials is a prerequisite for the industrial implication of the process. In this regard, we show a highly reliable and economical method for the preparation of Ni-colloidal nanoparticles derived thin-film nano-electrocatalysts to be employed as water oxidation catalysts. The colloidal Ni nano-catalysts are thoroughly characterized *via* UV-vis electronic absorption, FTIR, SEM, EDS, XRD, XPS and Raman analysis, revealing the successful fabrication of the electrocatalyst constituting metal hydroxides/oxides of varying oxidation states with incorporated boron in the catalytic film. Furthermore, the electrocatalytic aptitude has been extensively investigated following standard protocols. Electrocatalytic explorations such as CV, CPE, and CCE analysis demonstrate that all catalysts discussed here (annealed and un-annealed) showed remarkable catalytic activity and durability during long-term electrolysis. However, applying the post-heat treatments at a lower temperature range of 250 °C and 500 °C for a short time can further enhance the morphological features, mechanical stability, electrochemical efficiency and durability of the electrocatalytic materials. Our results demonstrate a new strategy of using candidly prepared colloidal NPs as a robust nano-electrocatalyst for energy conversion applications. One of the main advantages of using colloidal nanoparticles as the catalytic material is that carbon, boron and other heteroatom contents can be easily inserted into the catalytic film from carbonate, borate or other anionic buffer systems, thus making the thin film electrocatalyst very promising for the water oxidation reaction and energy conversion processes. This method can further pave the way to developed electrocatalytic materials comprising various combinations of binary and ternary metal oxides for running the water oxidation and other redox reactions at a much lower energy cost.

## Conflicts of interest

There are no conflicts to declare.

## Supplementary Material

RA-009-C9RA07388D-s001

## References

[cit1] Joya K. S., Ehsan M. A., Babar N.-U.-A., Sohail M., Yamani Z. H. (2019). J. Mater. Chem. A.

[cit2] Roger I., Symes M. D. (2016). J. Mater. Chem. A.

[cit3] Concepcion J. J., Jurss J. W., Brennaman M. K., Hoertz P. G., Patrocinio A. O. T., Murakami Iha N. Y., Templeton J. L., Meyer T. J. (2009). Accounts Chem. Res..

[cit4] Gagliardi C. J., Vannucci A. K., Concepcion J. J., Chen Z., Meyer T. J. (2012). Energy Environ. Sci..

[cit5] Gray H. B. (2009). Nat. Chem..

[cit6] Cook T. R., Dogutan D. K., Reece S. Y., Surendranath Y., Teets T. S., Nocera D. G. (2010). Chem. Rev..

[cit7] Dinh C.-T., de Arquer F. P. G., Sinton D., Sargent E. H. (2018). ACS Energy Lett..

[cit8] Ardizzone S., Fregonara G., Trasatti S. (1990). Electrochim. Acta.

[cit9] Trasatti S. (1994). Electrochem. Novel Mater..

[cit10] Wu J.-X., He C.-T., Li G.-R., Zhang J.-P. (2018). J. Mater. Chem. A.

[cit11] Liang H., Meng F., Cabán-Acevedo M., Li L., Forticaux A., Xiu L., Wang Z., Jin S. (2015). Nano Lett..

[cit12] Louie M. W., Bell A. T. (2013). J. Am. Chem. Soc..

[cit13] Li Y., Hasin P., Wu Y. (2010). Adv. Mater..

[cit14] Suntivich J., May K. J., Gasteiger H. A., Goodenough J. B., Shao-Horn Y. (2011). Science.

[cit15] Gerken J. B., McAlpin J. G., Chen J. Y., Rigsby M. L., Casey W. H., Britt R. D., Stahl S. S. (2011). J. Am. Chem. Soc..

[cit16] Gorlin Y., Jaramillo T. F. (2010). J. Am. Chem. Soc..

[cit17] Gong M., Li Y., Wang H., Liang Y., Wu J. Z., Zhou J., Wang J., Regier T., Wei F., Dai H. (2013). J. Am. Chem. Soc..

[cit18] Yeo B. S., Bell A. T. (2011). J. Am. Chem. Soc..

[cit19] Li Y., Hasin P., Wu Y. (2010). Adv. Mater..

[cit20] Gardner G. P., Go Y. B., Robinson D. M., Smith P. F., Hadermann J., Abakumov A., Greenblatt M., Dismukes G. C. (2012). Angew. Chem., Int. Ed..

[cit21] Gao M.-R., Xu Y.-F., Jiang J., Zheng Y.-R., Yu S.-H. (2012). J. Am. Chem. Soc..

[cit22] Cobo S., Heidkamp J., Jacques P.-A., Fize J., Fourmond V., Guetaz L., Jousselme B., Ivanova V., Dau H., Palacin S. (2012). Nat. Mater..

[cit23] Dincă M., Surendranath Y., Nocera D. G. (2010). Proc. Natl. Acad. Sci. U.S.A..

[cit24] Ma M., Liu K., Shen J., Kas R., Smith W. A. (2018). ACS Energy Lett..

[cit25] Joya K. S., Sinatra L., AbdulHalim L. G., Joshi C. P., Hedhili M. N., Bakr O. M., Hussain I. (2016). Nanoscale.

[cit26] Bayatsarmadi B., Zheng Y., Casari C. S., Russo V., Qiao S.-Z. (2016). Chem. Commun..

[cit27] Chen S., Duan J., Bian P., Tang Y., Zheng R., Qiao S. Z. (2015). Adv. Energy Mater..

[cit28] Yu X.-Y., Feng Y., Guan B., Lou X. W. D., Paik U. (2016). Energy Environ. Sci..

[cit29] Andersen N. I., Serov A., Atanassov P. (2015). Appl. Catal., B.

[cit30] Wu L., Li Q., Wu C. H., Zhu H., Mendoza-Garcia A., Shen B., Guo J., Sun S. (2015). J. Am. Chem. Soc..

[cit31] Joya K. S., Joya Y. F., De Groot H. J. (2014). Adv. Energy Mater..

[cit32] Han L., Dong S., Wang E. (2016). Adv. Mater..

[cit33] Chen S., Zhao Y., Sun B., Ao Z., Xie X., Wei Y., Wang G. (2015). ACS Appl. Mater. Interfaces.

[cit34] Kalasina S., Phattharasupakun N., Maihom T., Promarak V., Sudyoadsuk T., Limtrakul J., Sawangphruk M. (2018). Sci. Rep..

[cit35] Li F., Bai L., Li H., Wang Y., Yu F., Sun L. (2016). Chem. Commun..

[cit36] McCrory C. C., Jung S., Peters J. C., Jaramillo T. F. (2013). J. Am. Chem. Soc..

[cit37] Hall D. S., Lockwood D. J., Bock C., MacDougall B. R. (2015). Proc. R. Soc. A.

[cit38] Aggarwal P., Goswami A. (1961). J. Phys. Chem..

[cit39] Croguennec L., Pouillerie C., Delmas C. (2000). J. Electrochem. Soc..

[cit40] Fayemi O. E., Adekunle A. S., Ebenso E. E. (2015). J. Biosens. Bioelectron..

[cit41] Zhang Z., Zhao Y., Zhu M. (2006). Appl. Phys. Lett..

[cit42] Joya K. S., Babar N.-U.-A., Gilani S. R., Yasmeen F., Sarfaraz M., Ikram S., Colak S. G., Ocakoglu K., Ince M. (2018). ChemistrySelect.

[cit43] Chen Y., Kang J., Chen B., Gao B., Liu L., Liu X., Wang Y., Wu L., Yu H., Wang J. (2012). J. Phys. D: Appl. Phys..

[cit44] Anantharaj S., Ede S., Karthick K., Sankar S. S., Sangeetha K., Karthik P., Kundu S. (2018). Energy Environ. Sci..

[cit45] Kauffman D. R., Alfonso D., Tafen D. N., Lekse J., Wang C., Deng X., Lee J., Jang H., Lee J.-s., Kumar S. (2016). ACS Catal..

[cit46] Chen S., Duan J., Ran J., Jaroniec M., Qiao S. Z. (2013). Energy Environ. Sci..

[cit47] Chen S., Duan J., Jaroniec M., Qiao S. Z. (2013). Angew. Chem., Int. Ed..

[cit48] Zhao Z., Wu H., He H., Xu X., Jin Y. (2014). Adv. Funct. Mater..

