# Supplementary material for: Surface-assembled non-noble metal nanoscale Ni-colloidal thin-films as efficient electrocatalysts for water oxidation
Source: RSC Adv. 2019 Nov 14;9(64):37274–86. doi: 10.1039/c9ra07388d (PMC9075524; doi:10.1039/c9ra07388d)
Supplement: RA-009-C9RA07388D-s001 [file RA-009-C9RA07388D-s001.pdf]

Electronic Supplementary Material (ESI) for RSC Advances.
This journal is © The Royal Society of Chemistry 2019

Supporting Information

Surface-Assembled Non-Noble Metal Nanoscale Ni-Colloidal Thin-Films as Efficient Electrocatalyst for Water Oxidation

Noor-Ul-Ain Babar, Ayesha Saddiqa, Laraib Nisar, Syeda Robina Gilani, Khurram Saleem Joya*

MATERIALS

$Ni(NO_3)_2.6H_2O$ (99.99%), sodium hydroxide [NaOH (99.99%)], boric acid [$B(OH)_3$ (99.99%)], sodium bicarbonate [$NaHCO_3$ (99.99%)] sodium carbonate [Na_2CO_3 (99.99%)], potassium chloride [KCl (99.99%)], nitric acid (HNO_3) and sulfuric acid (H_2SO_4) were purchased from Aldrich and used as received without following any purification step. Methanol, ethanol, and acetone were obtained from Aldrich and were used after distillation. All the solutions involving water were made using ultrapure water (Milli-Q 18.2 MΩ cm, 2-4 ppb total organic contents).

MATERIALS CHARACTERIZATION

The average particle size and zeta potential of thus prepared metal colloidal nanoparticles (CNPs) are measured on Anton Paar's PSA analyzer. Fourier transform infrared spectroscopy (FTIR) spectrums are taken on Agilent Cary 630 FTIR spectrometer within the frequency range from 650 cm^{1-} to 4000 cm^{1-}. UV-Visible spectrums are recorded on Agilent Cary 60 UV-Visible spectrophotometer. To investigate the surface morphology and structure of electrocatalyst films supported on FTO, the scanning electron micrograph images are taken using the Nova Nano SEM microscope (NOVA FEI SEM-450 equipped with EDX detector). The surface composition of thin film nano-electrocatalyst is evaluated using energy dispersive X-Ray spectroscopy on a NOVA FEI SEM-450 equipped with EDX detector. The crystallinity and phase of the thin film metal-hydroxide/oxides materials are identified by powder X-Ray diffraction spectroscopy using Rigaku-Dmax 3C diffractometer (Rigaku Corp Tokyo Japan) with Cu-Kα (λ_{max}= 1.54056) radiations. RAMAN analysis is carried out on iRaman 532 nm RAMAN spectrometer (SN.17003)/iRaman. To exactly determine chemical nature and oxidation states of catalyst X-ray photoelectron spectroscopy is undertaken on a Versa-ProbeⅢ XPS Instrument (PHI 5000, ULVAC-PHI) X resource:100u25w15KV.

ELECTROCHEMICAL MEASUREMENTS

Exchange current density [(mA cm^{-2})]

Exchange current density is calculated considering charge transfer resistance at electrode-electrolyte interphase using the following equation S1 [1-2]

$$Jexc = \frac{RT}{\mathrm{nAF}\theta}$$

Where,
R= universal gas constant 8.314 J ($kg.m^2.s^{-2}$)/K. mol, T= is temperature 298 K, n= number of electrons which is 4, F= faraday constant 96485 C (A.s)/mol, θ= charge transfer resistance Ω ($kg.m^2.s^{-3}.A^{-2}$), A= geometrical area of working electrode.

Ni-CNPs/FTO

=8.314 J /K. mol × 298 K / 4 × 96485 C / mol × 64 Ω × 1 cm^2

= 0.10 mA cm^{2-}

Ni-CNPs/FTO_{250}

=8.314 J /K. mol × 298 K / 4 × 96485 C / mol × 42 Ω × 1 cm^2

= 0.15 mA cm^{2-}

Ni-CNPs/FTO$_{500}$

$$=8.314 \text{ J /K. mol} \times 298 \text{ K} / 4 \times 96485 \text{ C / mol} \times 18\ \Omega \times 1 \text{ cm}^2$$

$$= 0.35 \text{ mA cm}^{2-}$$

Table S1. Thin film nano-electrocatalysts derived from Ni-CNPs investigated along with their preparation conditions

Sr.No.	Catalyst	Precursors	Deposition conditions
1	**Ni-CNPs/FTO**	1 mL of 0.1 M $Ni(NO_3)_2.6H_20$ and 0.1 mL of 0.2 M $B(OH_3)$ solution	Ni-CNPs deposited on FTO by drop casting and air dried
2	**Ni-CNPs/FTO_{250}**	1 mL of 0.1 M $Ni(NO_3)_2.6H_20$ and 0.1 mL of 0.2 M $B(OH_3)$ solution	Ni-CNPs deposited on FTO by drop casting and annealed at 250 ^{O}C for 1 hour
3	**Ni-CNPs/FTO_{500}**	1 mL of 0.1 M $Ni(NO_3)_2.6H_20$ and 0.1 mL of 0.2 M $B(OH_3)$ solution	Ni-CNPs deposited on FTO by drop casting and annealed at 500 ^{O}C for 1 hour

Table S2. EDS elemental analysis for nano-electrocatalysts derived from M-CNPs

Sr. No.	Catalysts	Elements Composition by Wt %			
		C	Ni	Co	O
**1**	**Ni-CNPs/FTO**	3.0	6.9	-	39.14
**2**	**Ni-CNPs/FTO_{250}**	4.9	30.1	-	49.9
**3**	**Ni-CNPs/FTO_{500}**	3.6	39.45	-	39.14

Table S3. OER parameters investigated for each catalyst coated on FTO in 0.1 M NaOH electrolyte solution

Sr. No	Catalyst	Onset potential V (vs. RHE)	η (mV) (vs. RHE)	η (mV) at 10 mA cm^{2-}	Peak current density (mA cm^{2-})	Peak potential V (vs. RHE) at peak current density
1	**Ni-CNPs/FTO**	1.55 ± 0.02	320	420	>28	1.82
2	**Ni-CNPs/FTO$_{250}$**	1.53 ± 0.03	300	320	>33	1.71
3	**Ni-CNPs/FTO$_{500}$**	1.48 ± 0.01	250	320	>46	1.71

Table S4. Comparative study of electrocatalytic activity of Ni-CNPs derived electrocatalysts on various substrates

Sr. No	Catalyst/Substrate	Onset potential V (vs. RHE)	Peak current density (mA cm^{2-})	Peak potential V (vs. RHE) at peak current density	Charge transfer resistance (Ω) at 350 mV
1	**Ni-CNPs/FTO**	1.55	>28	1.82	64
2	**Ni-CNPs/FTO_{250}**	1.53	>33	1.71	42
3	**Ni-CNPs/FTO_{500}**	1.48	>46	1.71	18
4	**Ni-CNPs/NF**	1.52	>28	1.79	13
5	**Ni-CNPs/NF_{250}**	1.47	>122	1.81	5
6	**Ni-CNPs/NF_{500}**	1.47	>140	1.63	4
7	**Ni-CNPs/CC**	1.54	>47	1.71	10
8	**Ni-CNPs/CC_{250}**	1.49	>50	1.70	7
9	**Ni-CNPs/CC_{500}**	1.47	>50	1.66	5
10	**Bare FTO**	-	>2.2	1.8	1,935
11	**Bare NF**	-	>40	2.0	65
12	**Bare CC**	-	>9	1.93	180

Table S5. Kinetic parameters investigated for each catalyst

Sr.No	Catalyst	Tafel Slope (mV dec^{1-})	R_{CT} (Ω)	Mass Activity (mA cm^{2-}) @0.35 V	ECSA (cm^2)	Exchange current density (mA cm^{2-})	TOF (s^{1-}) @0.35 v
1	**Ni-CNPs/FTO**	92	64	14.6	0.36	0.1	0.18
2	**Ni-CNPs/FTO$_{250}$**	74	42	41	27.4	0.15	0.45
3	**Ni-CNPs/FTO$_{500}$**	66	18	56.3	27.2	0.35	0.79

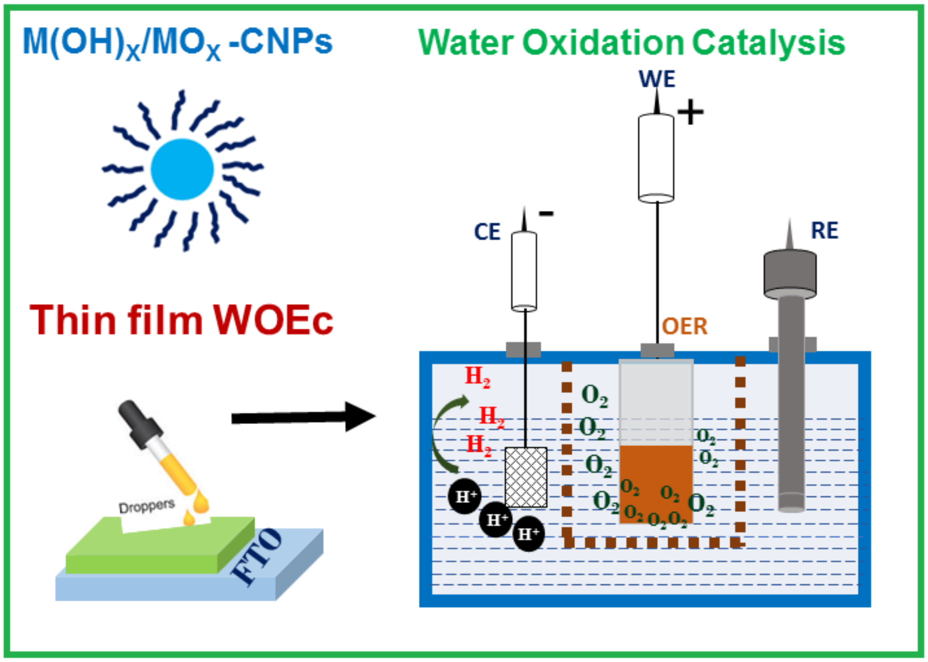

Scheme S1. Representative for preparing nano-electrocatalyst derived from easily made Ni-CNPs as an anode water for water oxidation catalysis.

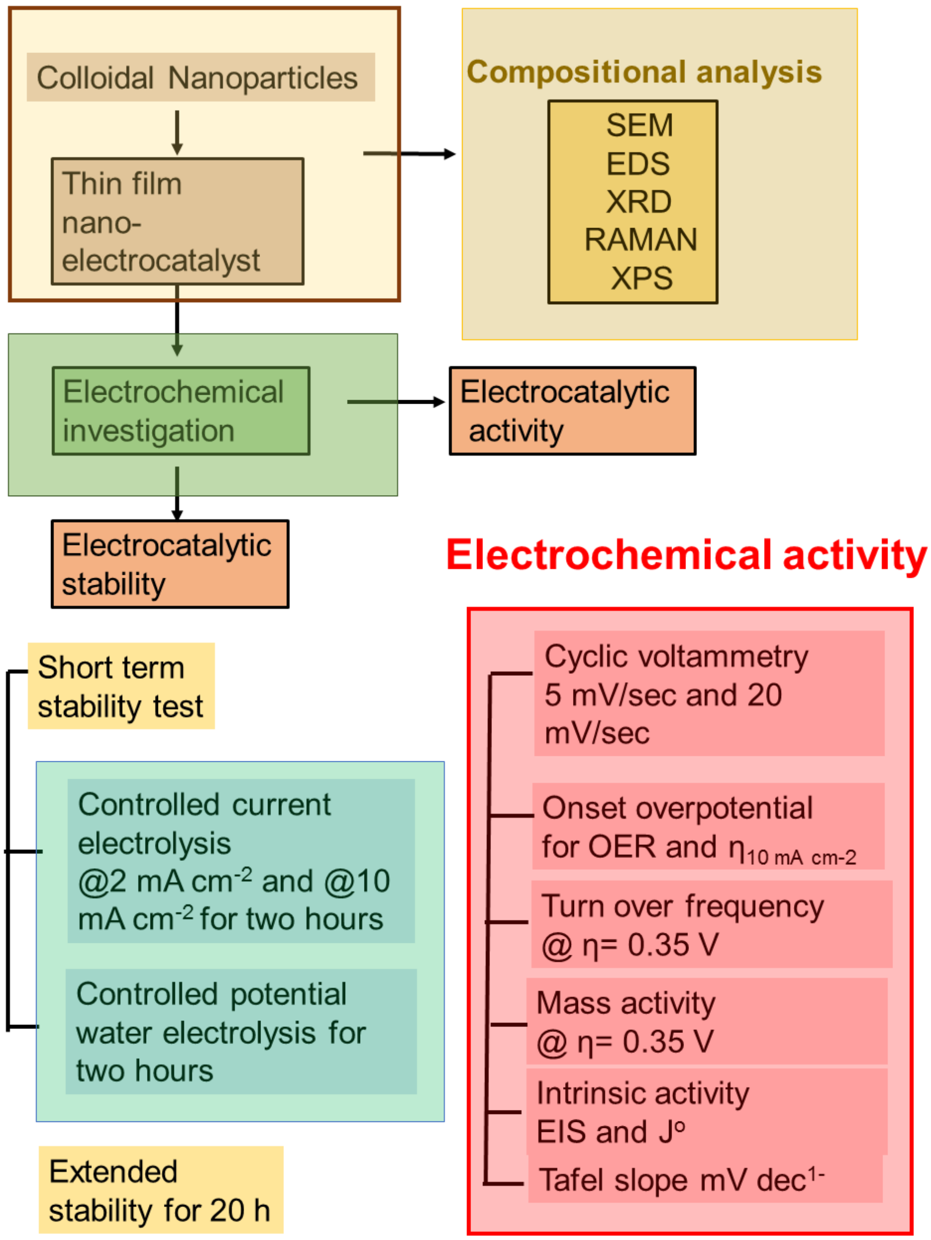

Scheme S2. Protocol for measuring electrocatalytic activity, electrochemical kinetics and durability for OER under mild conditions using M-CNPs derived nano-electrocatalysts coated on FTO substrate.

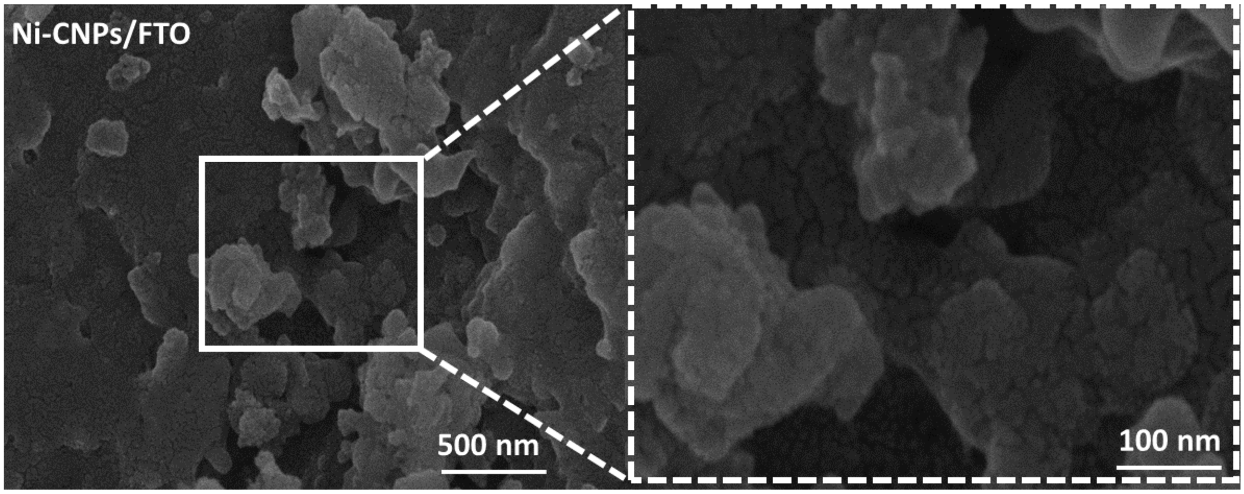

Figure S1. Scanning electron micrograph (SEM) image for Ni-CNPs/FTO prepared in borate buffer (pH≈10) **(left)** overview image **(right)** high resolution image.

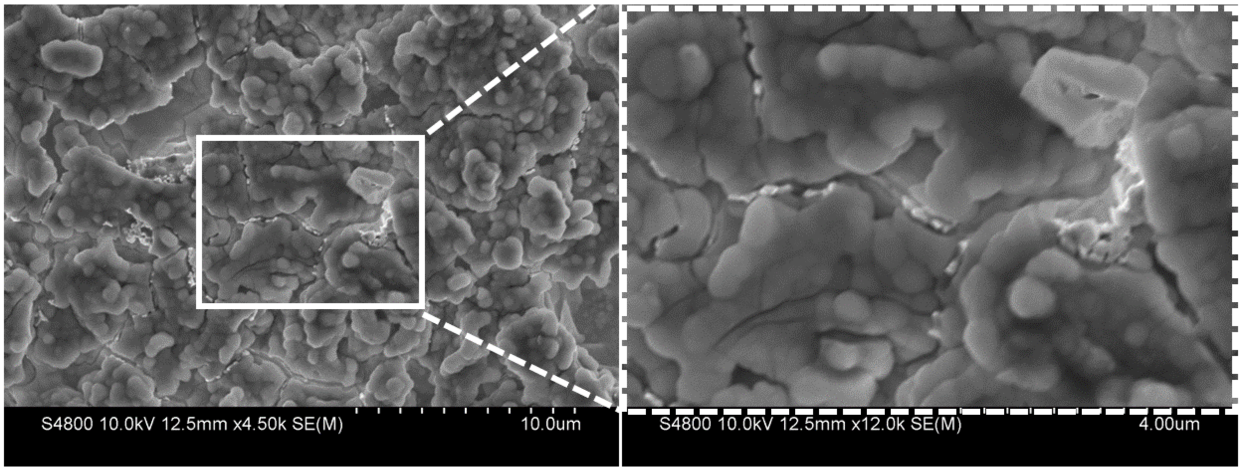

Figure S2. Scanning electron micrograph (SEM) image for Ni-CNPs/FTO prepared in borate buffer (pH≈11) **(left)** overview image **(right)** high resolution image.

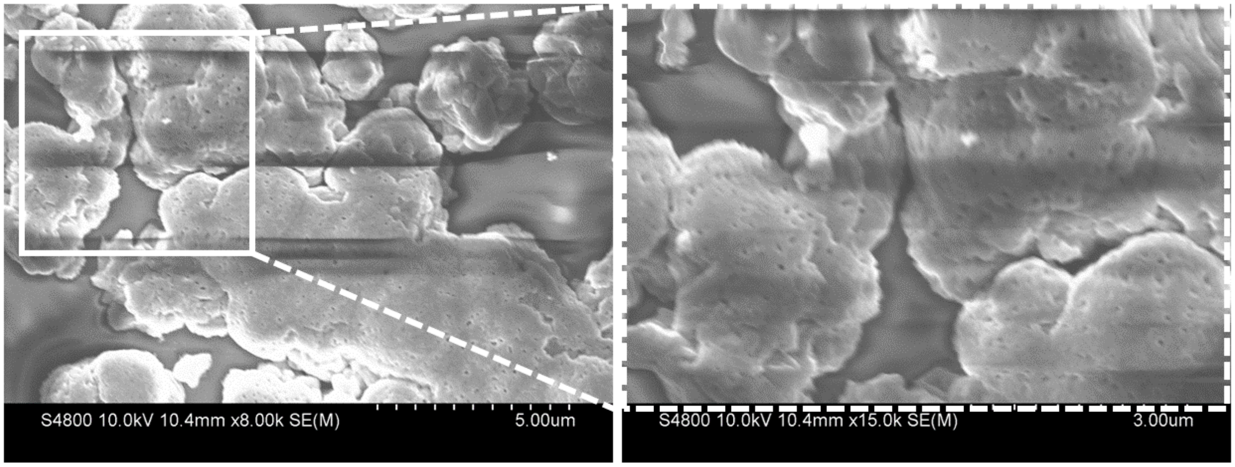

Figure S3. Scanning electron micrograph (SEM) image for Ni-CNPs/FTO prepared in borate buffer (pH≈12) **(left)** overview image **(right)** high resolution image.

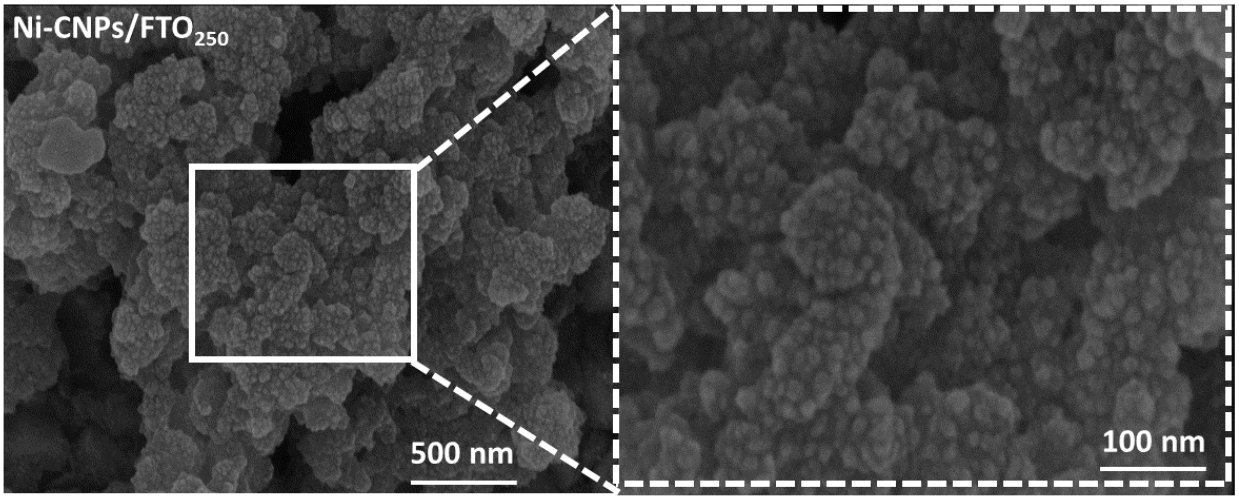

Figure S4. Scanning electron micrograph (SEM) image for Ni-CNPs/FTO_{250} **(left)** overview image **(right)** high resolution image.

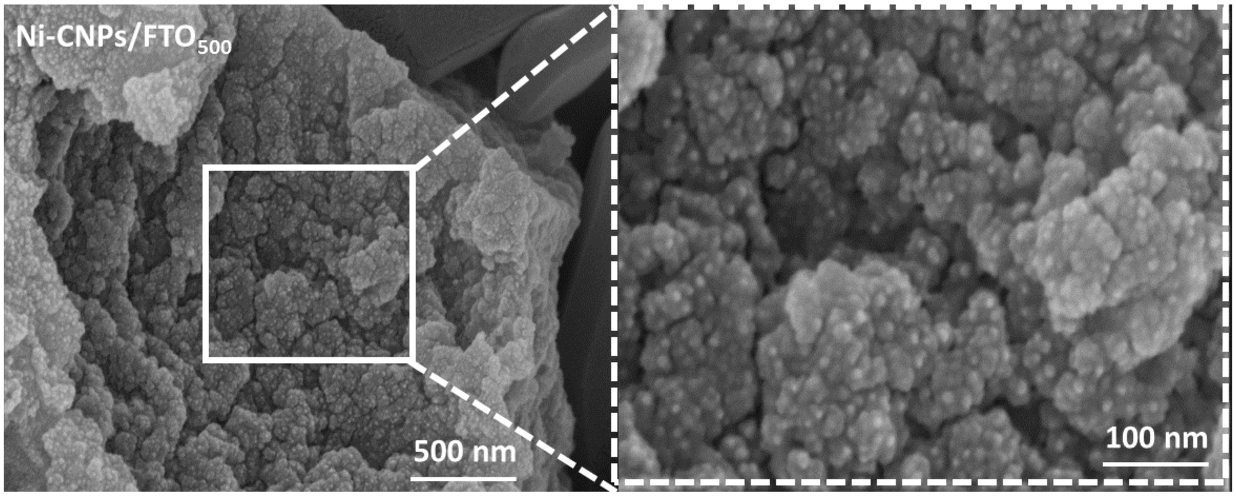

Figure S5. Scanning electron micrograph image (SEM) for Ni-CNPs/FTO_{500} **(left)** overview image **(right)** high resolution image.

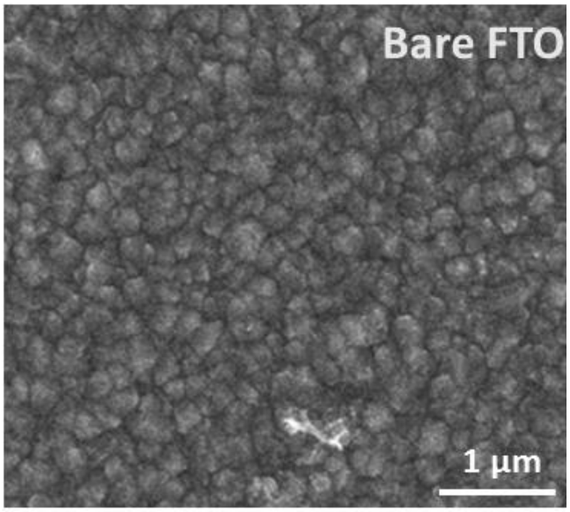

Figure S6. Scanning electron micrograph (SEM) image for bare FTO electrode without catalyst coating.

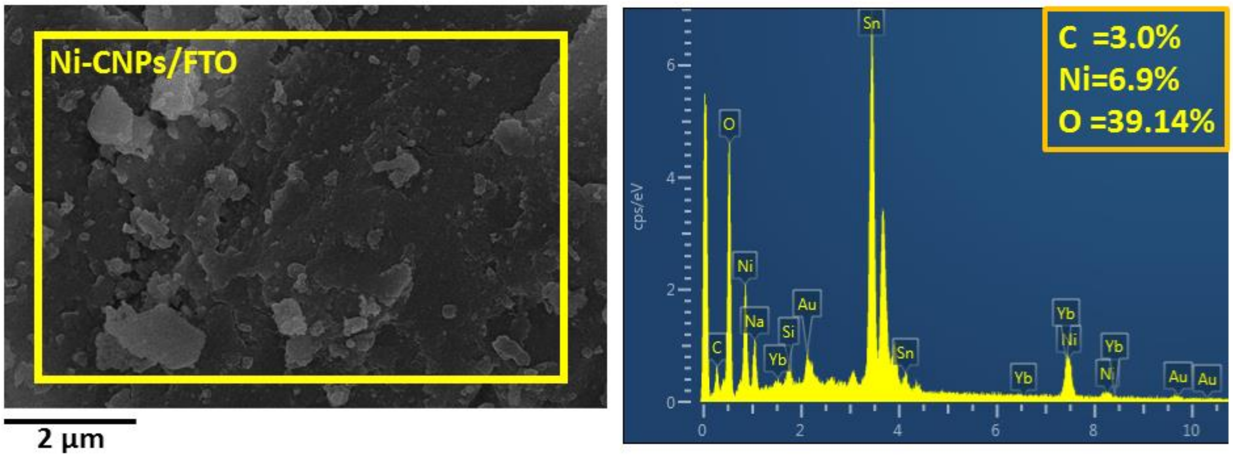

Figure S7. Energy dispersive X-ray (EDS) spectrum for Ni-CNPs/FTO.

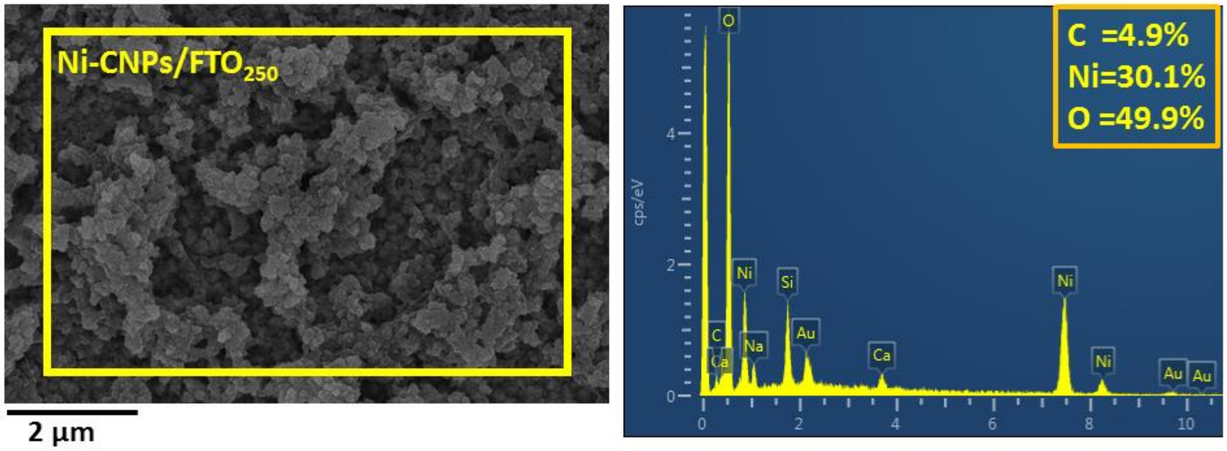

Figure S8. Energy dispersive X-ray (EDS) spectrum for Ni-CNPs/FTO_{250}.

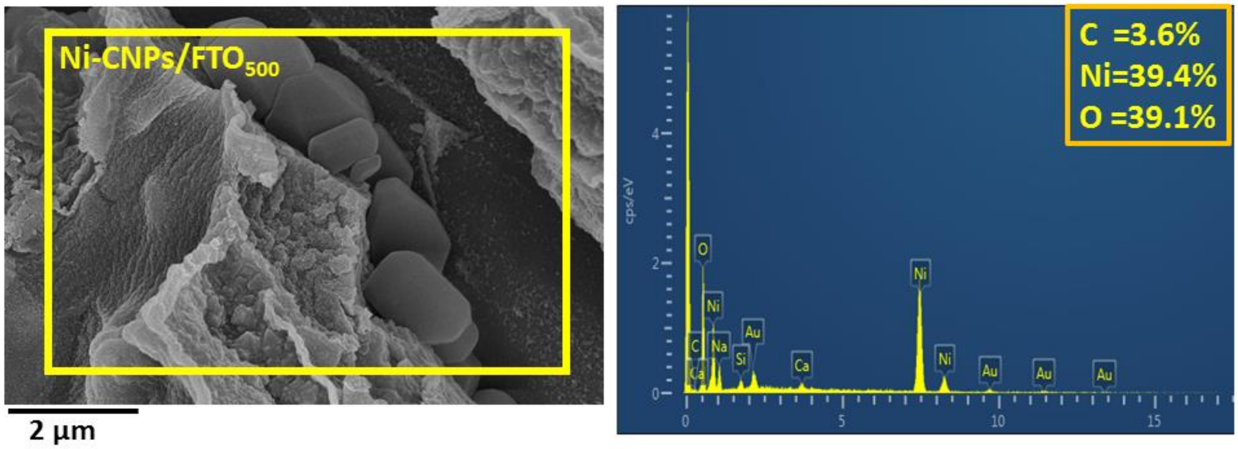

Figure S9. Energy dispersive X-ray (EDS) spectrum for Ni-CNPs/FTO_{500}.

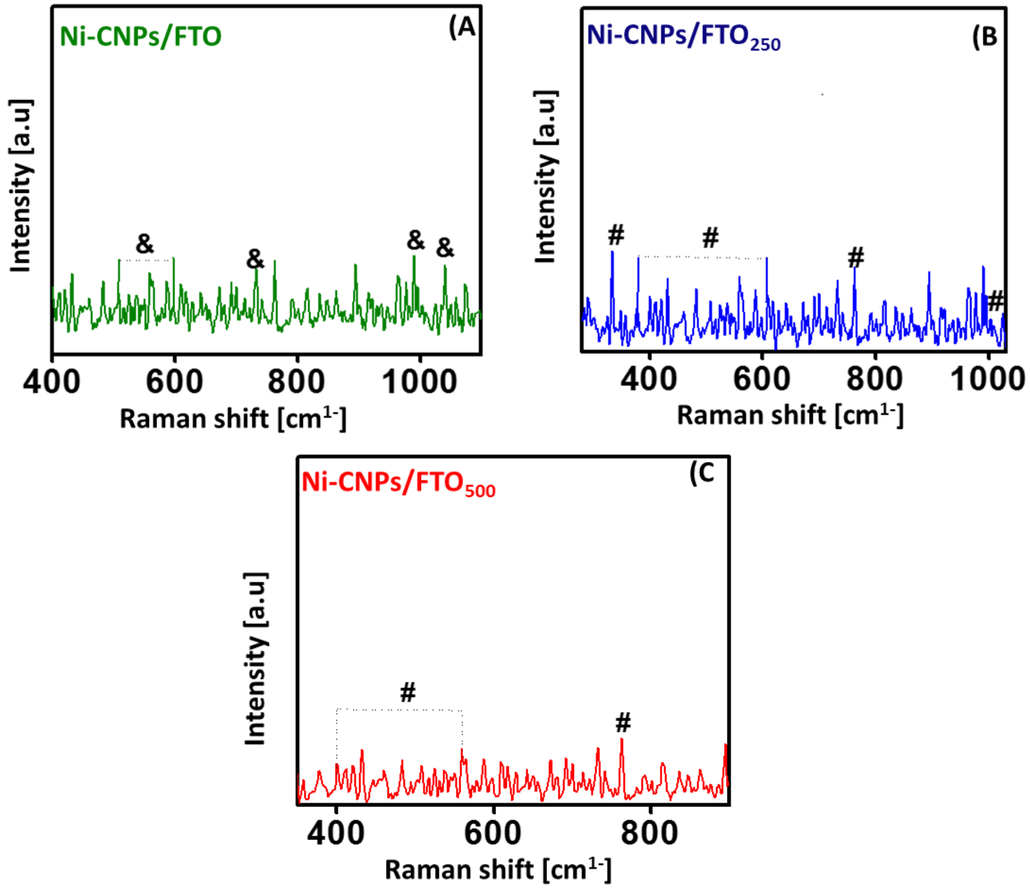

Figure S10. RAMAN spectrum for (a) Ni-CNPs/FTO (b) Ni-CNPs/FTO$_{250}$ (c) Ni-CNPs/FTO$_{500}$ where **&** and **#** represent $Ni(OH)_2$ and NiOx type structural features.

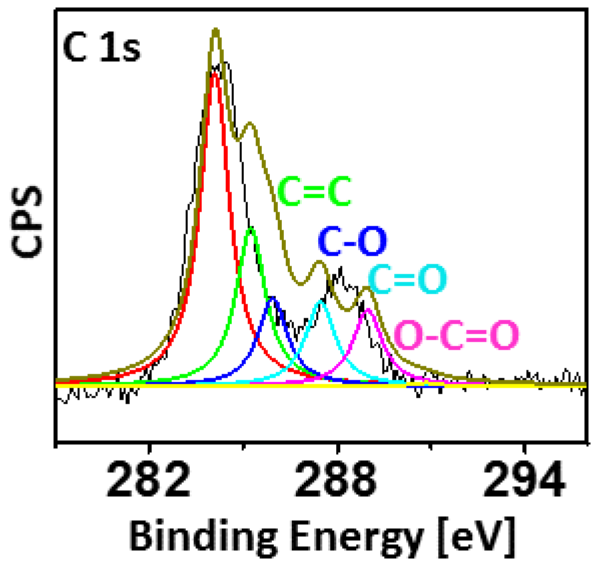

Figure S11. XPS spectrum for Ni-CNPs/FTO_{500} showing C1s (enlarged view).

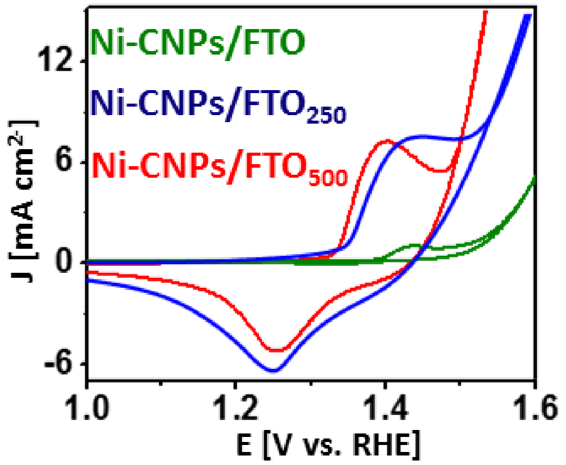

Figure S12. Representative cyclic polarization curves of OER at scan rate of 5 mV s^{1-} for (green) Ni-CNPs/FTO (blue) Ni-CNPs/FTO_{250} (red) Ni-CNPs/FTO_{500} in 0.1 M NaOH electrolyte solution (enlarged view).

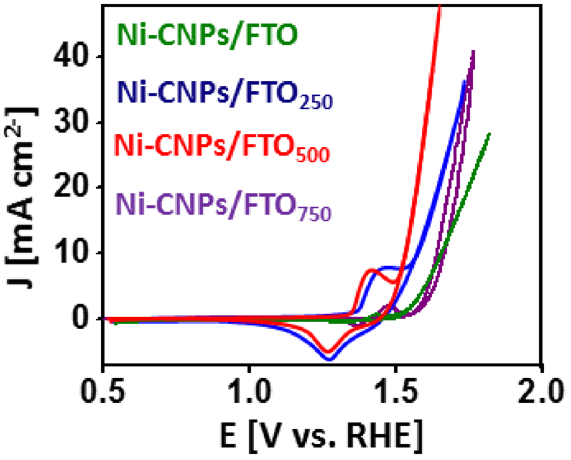

Figure S13. Representative cyclic polarization curves of OER at scan rate of 5 mV s^{1-} for (green) Ni-CNPs/FTO (blue) Ni-CNPs/FTO_{250} (red) Ni-CNPs/FTO_{500} (purple) Ni-CNPs/FTO_{750} in 0.1 M NaOH electrolyte solution.

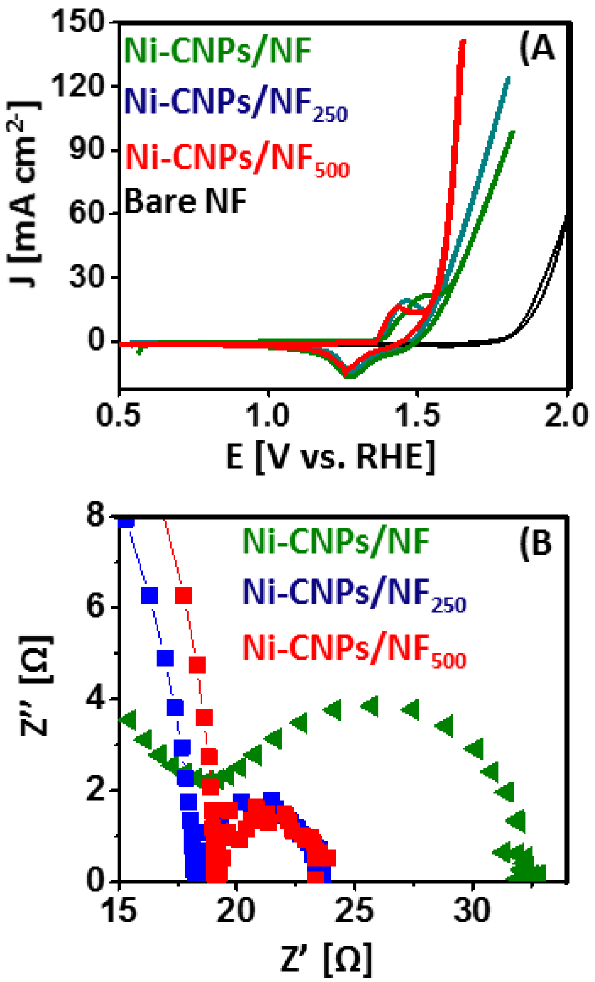

Figure S14. a) Representative cyclic polarization curves of OER at scan rate of 5 mV s^{1-} for (green) Ni-CNPs/NF (blue) Ni-CNPs/NF_{250} (red) Ni-CNPs/NF_{500} **b)** representative Nyquist plots for Ni-based nano-electrocatalysts coated on NF (NF=nickel foam, exposed area 1 cm^2) at applied potential of 1.58 V vs. RHE in 0.1 M NaOH electrolyte solution.

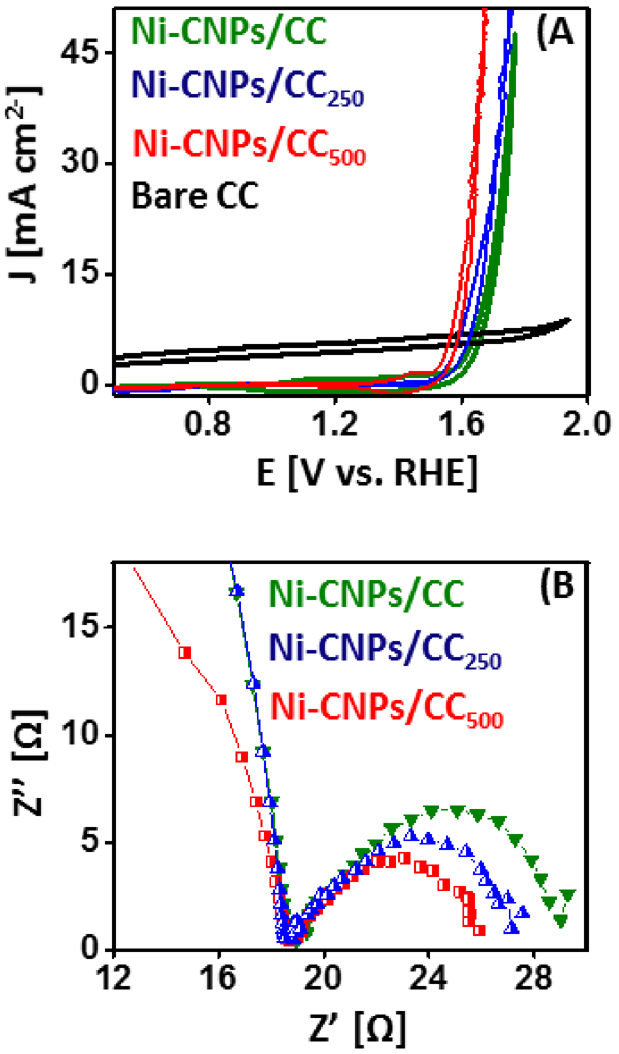

Figure S15. a) Representative cyclic polarization curves of OER at scan rate of 5 mV s^{1-} for (green) Ni-CNPs/CC (blue) Ni-CNPs/CC_{250} (red) Ni-CNPs/CC_{500} **b)** representative Nyquist plots for Ni-based nano-electrocatalysts coated on CC (CC=carbon cloth, exposed area 1 cm^2) at applied potential of 1.58 V vs. RHE in 0.1 M NaOH electrolyte solution.

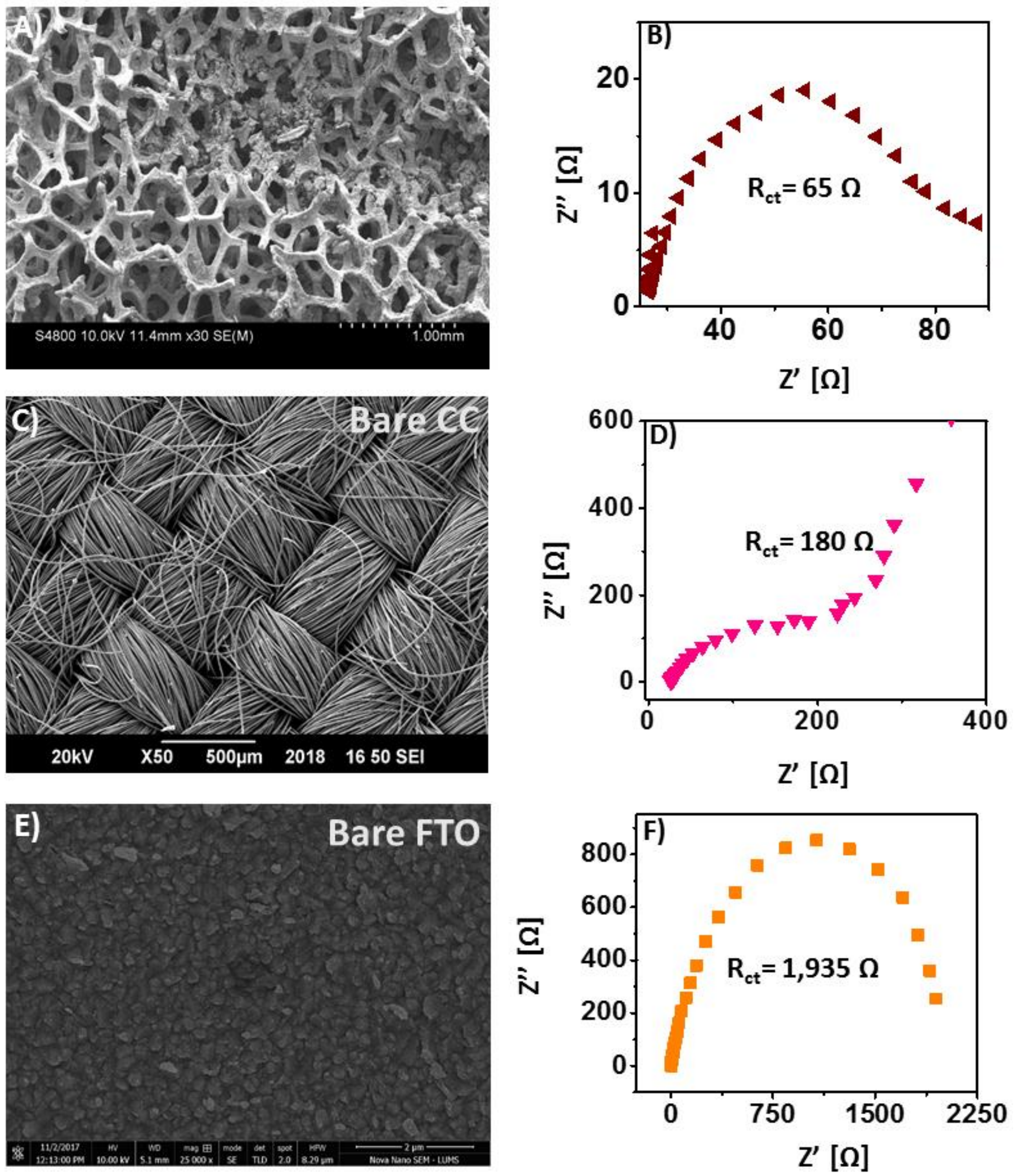

Figure S16. Representative scanning electron micrograph images and Nyquist plots for OER at applied potential of 450 mV in 0.1 M NaOH electrolyte solution for **(a-b)** bare nickel foam electrode **(c-d)** bare carbon cloth **(e-f)** bare FTO electrode.

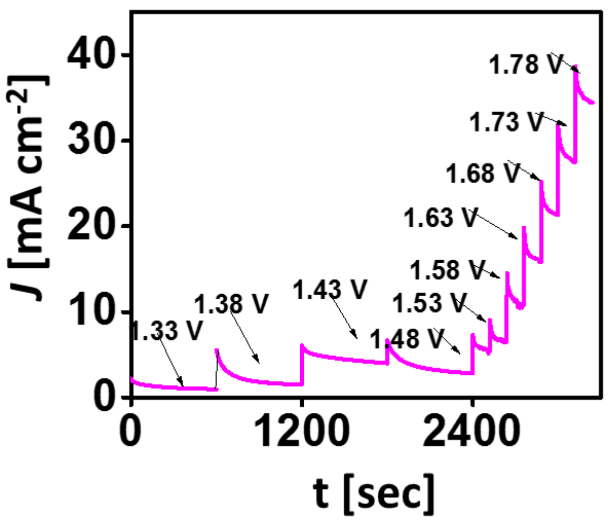

Figure S17. Representative multistep CPE presenting current density profile for consecutive potential increments from 1.33 V vs. RHE to 1.78 V vs. RHE for **Ni-CNPs/FTO$_{500}$** in 0.1 M NaOH electrolyte solution.

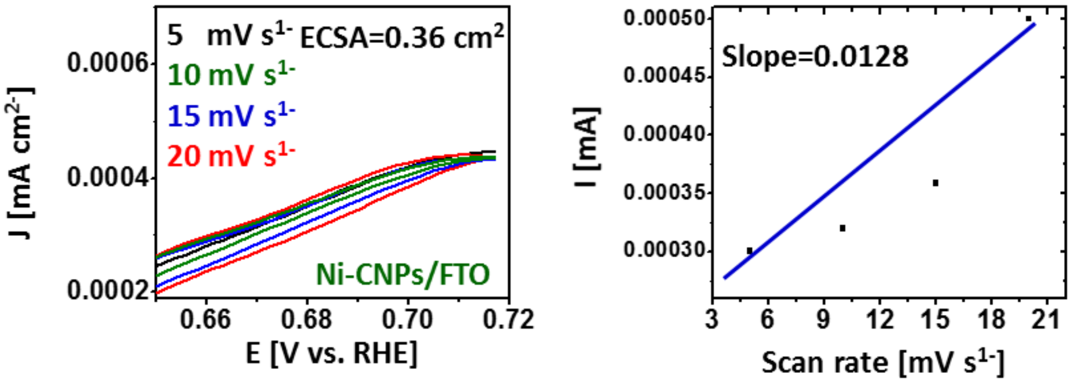

Figure S18. Double layer capacitance measurements for determining electrochemically active surface area (ECSA) of Ni-CNPs/FTO type electrocatalyst in 0.1 M NaOH electrolyte solution. (left) cyclic voltammogram measured at various scan rates such as from 5 mV s^{1-} to 20 mV s^{1-} in the non-faradaic regions of polarization curve, where all obtained current is supposed to be due to capacitive charging only; (right) charging current measured at the fixed potential of 0.69 V (vs. RHE) ultimately plotted as a function of scan rate where slope giving the value of double layer capacitance such as 0.0128 mF and ECSA is found to be 0.36 cm^2 (the geometrical area of working electrode is 1 cm^2).

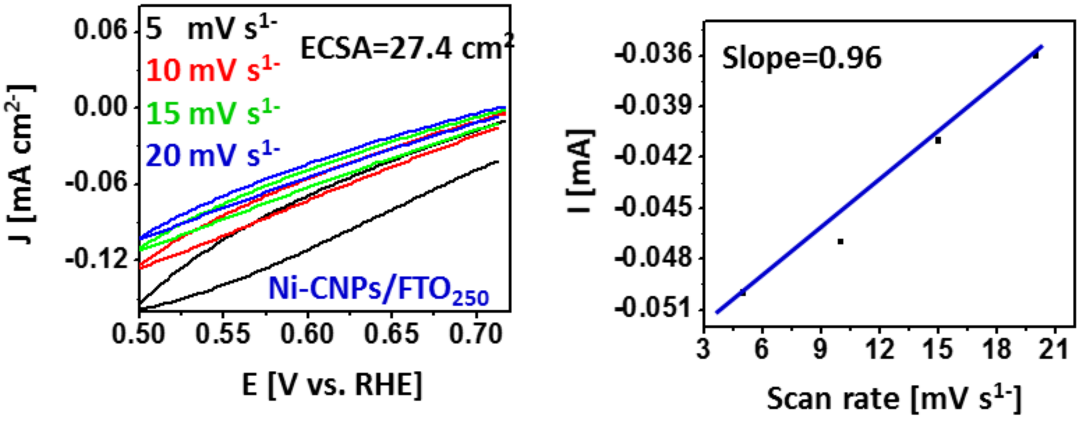

Figure S19. Double layer capacitance measurements for determining electrochemically active surface area (ECSA) of Ni-CNPs/FTO_{250} type electrocatalyst in 0.1 M NaOH electrolyte solution. (left) cyclic voltammogram measured at various scan rates such as from 5 mV s^{1-} to 20 mV s^{1-} in the non-faradaic regions of polarization curve, where all obtained current is supposed to be due to capacitive charging only; (right) charging current measured at the fixed potential of 0.60 V (vs. RHE) ultimately plotted as a function of scan rate where slope giving the value of double layer capacitance 0.96 mF and ECSA is found to be 27.4 cm^2 (the geometrical area of working electrode is 1 cm^2)

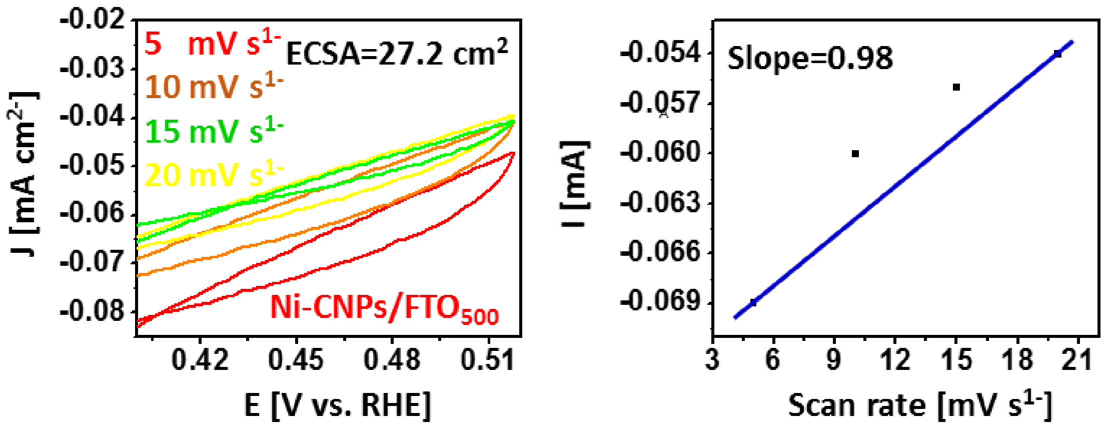

Figure S20. Double layer capacitance measurements for determining electrochemically active surface area (ECSA) of Ni-CNPs/FTO_{500} type electrocatalyst in 0.1 M NaOH electrolyte solution. (left) cyclic voltammogram measured at various scan rates such as from 5 mV s^{1-} to 20 mV s^{1-} in the non-faradaic regions of polarization curve, where all obtained current is supposed to be due to capacitive charging only; (right) charging current measured at the fixed potential of 0.46 V (vs. RHE) ultimately plotted as a function of scan rate where slope giving the value of double layer capacitance 0.98 mF and ECSA is found to be 27.2 cm^2 (the geometrical area of working electrode is 1 cm^2).

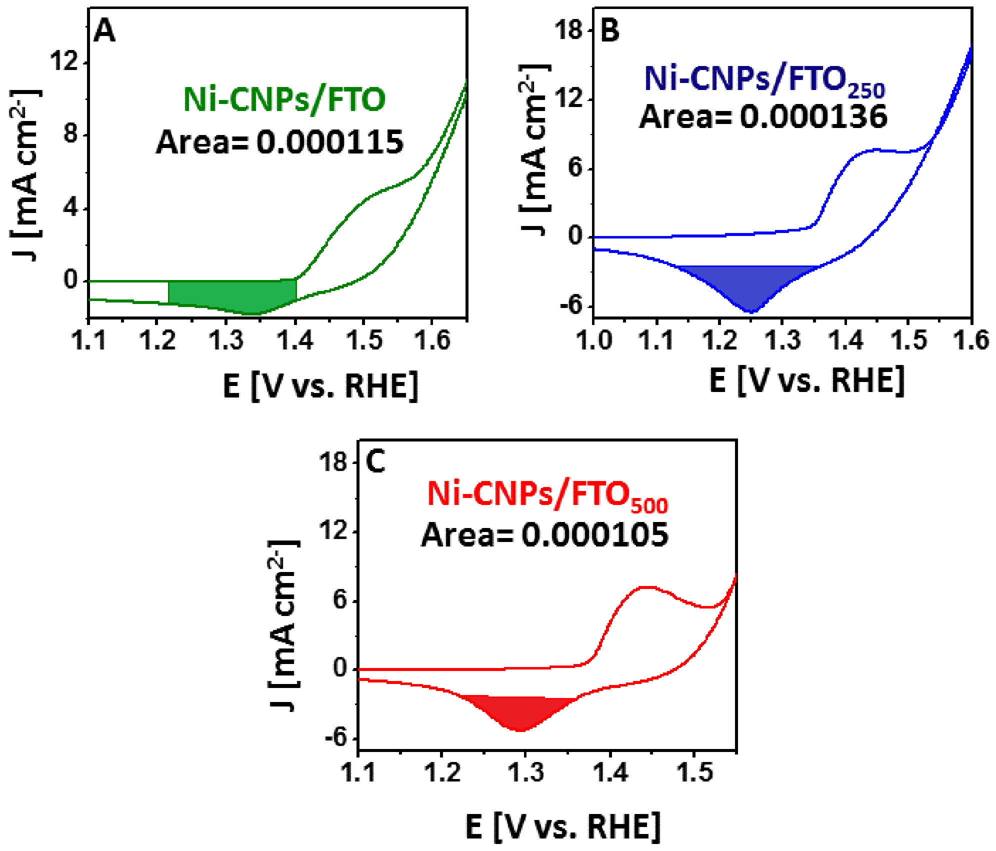

Figure S21. Area under the reduction peak of $Ni^{+2/+3}$ redox couple for **(a)** Ni-CNPs/FTO **(b)** Ni-CNPs/FTO_{250} **(c)** Ni-CNPs/FTO_{500} directly calculated from polarization curve within specific potential vertex in 0.1 M NaOH electrolyte solution.

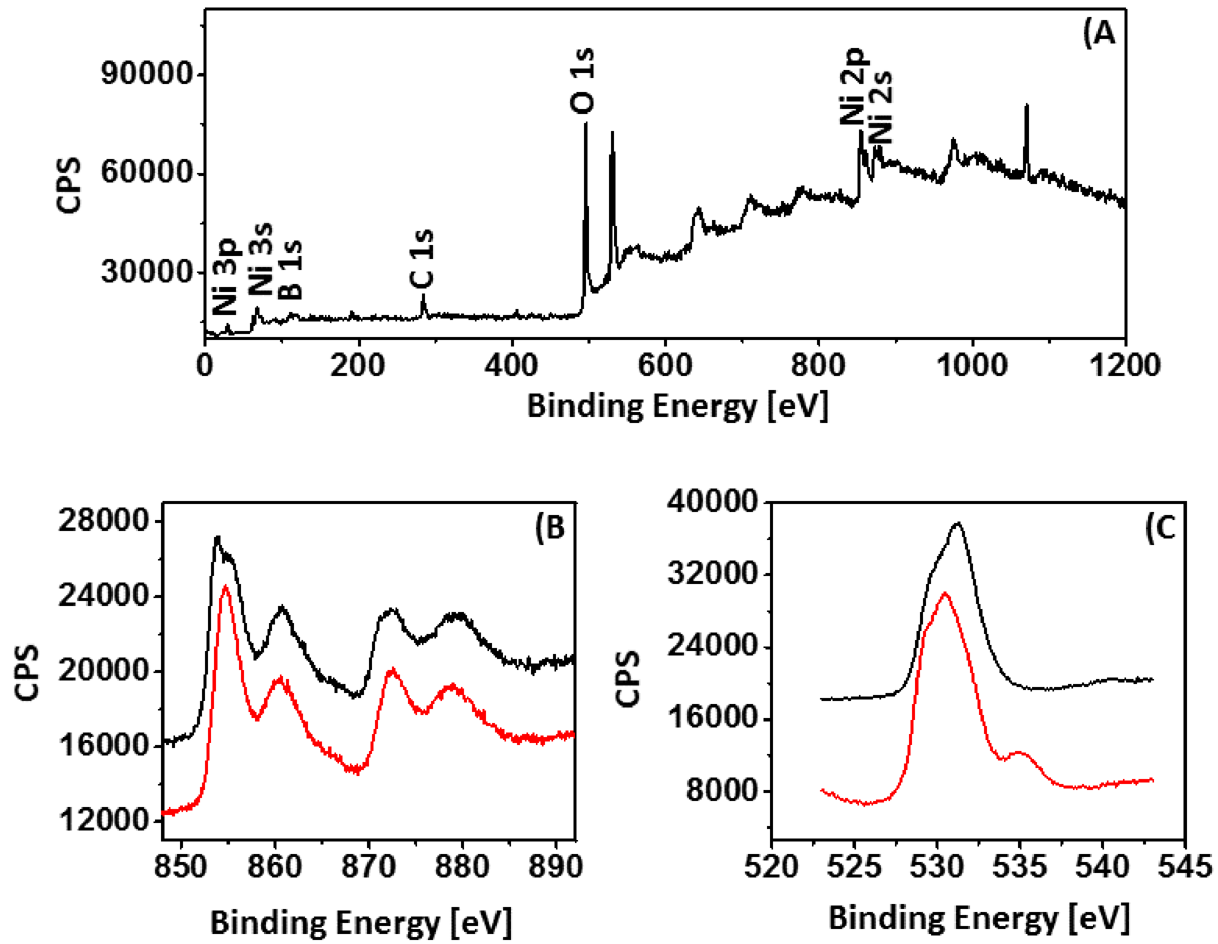

Figure 22. XPS spectrum for Ni-CNPs/FTO_{500} type nano-electrocatalyst after long term CPE analysis showing (a) survey spectrum; high-resolution XPS for (B) Ni and (C) oxygen (red is before catalysis and black is after catalysis.

Determination of surface concentration of Ni-CNPs/FTO from CV curve

Charge passed calculated by integrating the area (0.000115757) under reduction peak from potential vs. current curve is 0.00578787 Coulomb.
Then, no of electrons = $(0.00578787\ C) / (1.602 \times 10^{-19}\ C)$
$= 3.612903 \times 10^{16}$ electrons

The surface concentration of the Ni atom on the electrode is determined by diving with no of electrons involved in the redox reaction which is 1 for $Ni^{+3/+2}$, therefore,

Surface Conc. of atoms= $3.612903 \times 10^{16} / 1$
$=3.612903 \times 10^{16}$ atoms

Determination of TOF from integrated OER CV curve

We calculated turn over frequency (TOF) at 1.58 V (vs. RHE) (η=0.35 V) as specific potential value. Furthermore, TOF is calculated at various applied potential and the resulting TOF values are presented in Table S5.
Turn over frequency is calculated at various potential value considering the following equation S2 [2-3]

$$\mathrm{TOF} = \frac{\mathrm{I} \times \mathrm{NA}}{\mathrm{A} \times \mathrm{n} \times \mathrm{F} \times \Gamma}$$

Where,
I = current value at a specified potential (A)
N_A= Avogadro's constant which is 6.022×10^{23}
A = Geometrical area of the working electrode which is 1 cm^2
N= No of electrons
F= Faraday constant which is 96485 coulombs
Γ = surface concentration of atom on the electrode surface

TOF@ **1.51V**= $[(0.001) \times (6.022 \times 10^{23})] / [(1) \times (4) \times (96485) \times (0.003612903\times 10^{19})]$
$= 0.043188$ **s^{1-}**

TOF@ **1.53V**= $[(0.0014) \times (6.022 \times 10^{23})] / [(1) \times (4) \times (96485) \times (0.003612903\times 10^{19})]$

$= 0.060463$ **s^{1-}**

TOF@ **1.55V**= $[(0.0022) \times (6.022 \times 10^{23})] / [(1) \times (4) \times (96485) \times (0.003612903\times 10^{19})]$

$= 0.095014$ **s^{1-}**

TOF@ **1.57V**= $[(0.0029) \times (6.022 \times 10^{23})] / [(1) \times (4) \times (96485) \times (0.003612903\times 10^{19})]$

$= 0.125246$ $\mathbf{s^{1-}}$

TOF@ **1.58V**= $[(0.00432) \times (6.022 \times 10^{23})] / [(1) \times (4) \times (96485) \times (0.00939\times 10^{19})]$

$= 0.18$ $\mathbf{s^{1-}}$

TOF@ **1.59V**= $[(0.00457) \times (6.022 \times 10^{23})] / [(1) \times (4) \times (96485) \times (0.003612903\times 10^{19})]$

$= 0.19$ $\mathbf{s^{1-}}$

TOF@ **1.61V**= $[(0.00506) \times (6.022 \times 10^{23})] / [(1) \times (4) \times (96485) \times (0.003612903\times 10^{19})]$

$= 0.218532$ $\mathbf{s^{1-}}$

TOF@ **1.63V**= $[(0.00755) \times (6.022 \times 10^{23})] / [(1) \times (4) \times (96485) \times (0.003612903\times 10^{19})]$

$= 0.326071$ $\mathbf{s^{1-}}$

TOF@ **1.65V**= $[(0.00965) \times (6.022 \times 10^{23})] / [(1) \times (4) \times (96485) \times (0.003612903\times 10^{19})]$

$= 0.416766$ $\mathbf{s^{1-}}$

TOF@ **1.67V**= $[(0.0111) \times (6.022 \times 10^{23})] / [(1) \times (4) \times (96485) \times (0.003612903\times 10^{19})]$

$= 0.479389$ $\mathbf{s^{1-}}$

TOF@ **1.69V**= $[(0.01387) \times (6.022 \times 10^{23})] / [(1) \times (4) \times (96485) \times (0.003612903\times 10^{19})]$

$= 0.59902$ $\mathbf{s^{1-}}$

TOF@ **1.71V**= $[(0.01623) \times (6.022 \times 10^{23})] / [(1) \times (4) \times (96485) \times (0.003612903\times 10^{19})]$

$= 0.700944$ $\mathbf{s^{1-}}$

Following this standard pattern surface concentration of atoms and TOF values of all catalysts discussed in this study is calculated at various applied potentials and results are shown in Table S5.

Table S6. Turn over frequency calculations from OER polarization curves at various applied potentials for all catalysts.

**TOF (s^{1-})**	**Ni-CNPs/FTO**	**Ni-CNPs/FTO_{250}**	**Ni-CNPs/FTO_{500}**
TOF@1.51	0.04	0.25	0.15
TOF@1.53	0.06	0.31	0.28
TOF@1.55	0.09	0.34	0.37
TOF@1.57	0.12	0.42	0.61
TOF@1.58	0.18	0.45	0.79
TOF@1.59	0.19	0.47	0.87
TOF@1.61	0.21	0.62	1.14
TOF@1.63	0.32	0.73	1.44
TOF@1.65	0.41	0.80	1.62
TOF@1.67	0.47	0.91	1.84
TOF@1.69	0.59	0.98	2.07
TOF@1.71	0.70	1.05	2.24

Table S7. Short term stability outcomes evaluated for all catalysts during CCE and CPE experiments in 0.1 M NaOH electrolyte solution

Sr. No	Catalyst	Applied potential V (vs. RHE)	Overpotential (mV)	J [mA cm^{2-}] at t=0	J [mA cm^{2-}] at t=2 hours	$\eta_{@2mA\ cm^{2-}}$ [mV] at t=0 hour	$\eta_{@2mA\ cm^{2-}}$ [mV] at t=2 hours	$\eta_{@10mA\ cm^{2-}}$ [mV] at t=0 hour	$\eta_{@10mA\ cm^{2-}}$ [mV] at t=2 hours
1	**Ni-CNPs/FTO**	1.65	420	8.9	8.9	300	300	390	390
2	**Ni-CNPs/FTO_{250}**	1.55	320	9.8	10.5	250	250	380	370
3	**Ni-CNPs/FTO_{500}**	1.55	320	12.7	13.5	260	260	390	380

Table S8. Extended stability outcomes evaluated for all catalysts for 20 hours CPE experiments in 0.1 M NaOH electrolyte solution

**Sr. No**	**Catalyst**	**Applied potential V (vs RHE)**	**Overpotential (mV)**	**J at t=0**	**J at t=10 h**	**J at t=20h**	**%degradation after 10 h**	**%degradation after 20 h**	**Remarks**
1	**Ni-CNPs/FTO**	1.65	420	8.9	8.3	7.9	6	12	Stable
2	**Ni-CNPs/FTO$_{250}$**	1.55	320	9.8	11	12.8	Active	Active	Electro active
3	**Ni-CNPs/FTO$_{500}$**	1.55	320	12.7	13	13.6	Active	Active	Electro active

Reference

1. S. Anantharaj, S. Ede, K. Karthick, S. S. Sankar, K. Sangeetha, P. Karthik and S. Kundu, *Energy & Environmental Science*, 2018, **11**, 744-771.
2. N. U. A. Baber, K. S. Joya, M. A. Ehsan, M. Khan and M. Sharif, *ChemCatChem*, 2019, doi.org/10.1002/cctc.201900202.
3. K. S. Joya, M. A. Ehsan, N. U. A. Babar, M. Sohail and Z. H. Yamani, *Journal of Materials Chemistry A*, 2019, **7**, 9137-9144.
